# Plant Alkaloids as Promising Anticancer Compounds with Blood–Brain Barrier Penetration in the Treatment of Glioblastoma: In Vitro and In Vivo Models

**DOI:** 10.3390/molecules30071561

**Published:** 2025-03-31

**Authors:** Marcin Ożarowski, Tomasz M. Karpiński, Bogusław Czerny, Adam Kamiński, Agnieszka Seremak-Mrozikiewicz

**Affiliations:** 1 Department of Biotechnology, Institute of Natural Fibres and Medicinal Plants—National Research Institute, Wojska Polskiego 71b, 60-630 Poznań, Poland; 2 Chair and Department of Medical Microbiology, Poznań University of Medical Sciences, Rokietnicka 10, 60-806 Poznań, Poland; 3 Department of General Pharmacology and Pharmacoeconomics, Pomeranian Medical University in Szczecin, Żołnierska 48, 70-204 Szczecin, Poland; boguslaw.czerny@iwnirz.pl; 4 Institute of Natural Fibres and Medicinal Plants—National Research Institute, Wojska Polskiego 71b, 60-630 Poznań, Poland; 5 Department of Orthopaedics and Traumatology, Independent Public Clinical Hospital No. 1, Pomeranian Medical University in Szczecin, Unii Lubelskiej 1, 71-252 Szczecin, Poland; emluc@wp.pl; 6 Division of Perinatology and Women’s Disease, Poznań University of Medical Sciences, Polna 33, 60-535 Poznań, Poland; asm@data.pl; 7 Laboratory of Molecular Biology in Division of Perinatology and Women’s Diseases, University of Medical Sciences, Polna 33, 60-535 Poznań, Poland

**Keywords:** plant alkaloids, glioblastoma, blood–brain barrier, cell culture, preclinical studies, anticancer drugs from botanical source, mechanism of action, delivery systems

## Abstract

Glioblastoma (GBM) is one of the most invasive central nervous system tumors, with rising global incidence. Therapy resistance and poor prognosis highlight the urgent need for new anticancer drugs. Plant alkaloids, a largely unexplored yet promising class of compounds, have previously contributed to oncology treatments. While past reviews provided selective insights, this review aims to collectively compare data from the last decade on (1) plant alkaloid-based anticancer drugs, (2) alkaloid transport across the blood–brain barrier (BBB) in vitro and in vivo, (3) alkaloid mechanisms of action in glioblastoma models (in vitro, in vivo, ex vivo, and in silico), and (4) cytotoxicity and safety profiles. Additionally, innovative drug delivery systems (e.g., nanoparticles and liposomes) are discussed. Focusing on preclinical studies of single plant alkaloids, this review includes 22 botanical families and 28 alkaloids that demonstrated anti-GBM activity. Most alkaloids act in a concentration-dependent manner by (1) reducing glioma cell viability, (2) suppressing proliferation, (3) inhibiting migration and invasion, (4) inducing cell death, (5) downregulating Bcl-2 and key signaling pathways, (6) exhibiting antiangiogenic effects, (7) reducing tumor weight, and (8) improving survival rates. The toxic and adverse effect analysis suggests that alkaloids such as noscapine, lycorine, capsaicin, chelerythrine, caffeine, boldine, and colchicine show favorable therapeutic potential. However, tetrandrine, nitidine, harmine, harmaline, cyclopamine, cocaine, and brucine may pose greater risks than benefits. Piperine’s toxicity and berberine’s poor bioavailability suggest the need for novel drug formulations. Several alkaloids (kukoamine A, cyclovirobuxine D, α-solanine, oxymatrine, rutaecarpine, and evodiamine) require further pharmacological and toxicological evaluation. Overall, while plant alkaloids show promise in glioblastoma therapy, progress in assessing their BBB penetration remains limited. More comprehensive studies integrating glioma research and advanced drug delivery technologies are needed.

## 1. Introduction

### 1.1. Description of Glioblastoma

Glioblastoma multiforme (GBM) is the most common primary malignant brain tumor, accounting for 77–81% of all primary malignant tumors of the CNS [1]. This cancer subtype includes high-grade GBM and low-grade gliomas such as astrocytoma and oligodendroglioma [2,3]. GBM is one of the most invasive CNS tumors, characterized by high malignancy and poor prognosis [4,5]. Its global incidence is rising, with survival rates averaging only 15 months due to high recurrence and resistance to current treatments, including surgery, radiotherapy, and chemotherapy [6]. The therapy resistance and poor prognosis highlight the urgent need for new anticancer drugs.

According to the GLOBOCAN 2020 program, there were 19.3 million new cancer cases worldwide and 10 million cancer-related deaths [2]. Predictions indicate that by 2040, the number of cancer cases will rise to 28.4 million [7]. Between 1990 and 2016, the incidence of central nervous system (CNS) cancer increased by 17.3%, with East Asia reporting the highest number of cases in both sexes, followed by Western Europe. Additionally, China, the USA, and India had the highest numbers of CNS tumor cases, both malignant and non-malignant [2]. In 2020, an estimated 308,102 cases of nervous system cancers were reported [8].

In recent years, anticancer drug discovery strategies have evolved significantly, driven by advancements in understanding the molecular mechanisms of neoplastic transformation. Progress in drug discovery, development, and patient care has been fueled by insights into cancer biology, including glioblastoma [9], and detailed genomic data from resources like The Cancer Genome Atlas (TCGA) [10]. Oncology practice has also advanced, with treatments emerging for previously fatal cancers. However, major challenges persist, including drug toxicity, resistance, and the heterogeneous nature of cancer. Additionally, the narrow therapeutic index of anticancer drugs, adverse side effects, and limitations in drug delivery systems remain significant obstacles to effective treatment [11,12].

### 1.2. Review of Alkaloids (Group of Plant Metabolites and Group of Chemical Compounds Classified According to Chemical Structure)

Current knowledge of plant secondary metabolites allows us to state that a large group of alkaloids (organic chemical compounds containing nitrogen atoms in a heterocyclic ring structure) includes diverse categories of chemical molecules in plants. These alkaloids regulate plant growth and protect them against herbivores (e.g., nematodes and insects) [13,14,15] and various pathogens by reducing bacterial and fungal infestation, functioning as “botanical herbicides” [16,17,18]. Approximately 12,000 different alkaloids have been identified, primarily in higher plants [14,15]. Alkaloids are widely distributed in plants, fungi, bacteria, amphibians, insects, and marine animals. According to Pereira [19,20], more than 27,000 alkaloids have been characterized. These alkaloids belong to four major chemical groups:

I. Alkaloids with nitrogen heterocycles (true alkaloids)—six classes.
II. Protoalkaloids (alkaloids with nitrogen in the side chain)—two classes.
III. Pseudoalkaloids—one class of chemical compounds.
IV. Polyamines alkaloids.

The chemical diversity of alkaloids leads to a broad spectrum of biological and pharmacological activities in humans. These include antiviral, antifungal, and antibacterial properties, as well as anti-inflammatory, antimalarial, antihypertensive, and anti-diabetic effects. Additionally, alkaloids function as sedatives, painkillers, anti-neurodegenerative agents, and anticancer drugs [17,19,21,22]. Many alkaloids possess psychoactive properties, such as cocaine (from *Erythroxylum coca*) [23], nicotine (from *Nicotiana tabacum*) [24], atropine, scopolamine, hyoscyamine (from *Datura stramonium*, *Atropa belladonna*, *Mandragora officinarum*), cathine (from *Catha edulis*), reserpine (from *Rauwolfia serpentina*), mitragynine (from *Mitragyna speciosa*), octopamine (from *Ophiopogon japonicas*), arecoline (from *Areca catechu*), dimethyltryptamine, beta-carbolines, N-methyltryptamine (from *Psychotria viridis*), ibogaine (from *Tabernanthe iboga*), mescaline (from *Lophophora williamsii*) [25,26], and opium alkaloids, i.e., codeine and morphine (from *Papaver somniferum*) [23,27]. These psychoactive properties should be carefully considered in future studies on the design of alkaloid-based drugs for treating glioblastoma patients.

In current studies, 22 botanical families have been identified as sources of plant species containing alkaloids in publications focusing on anti-glioblastoma activities. These families and their representative species include the following:

- Apocynaceae (*Catharanthus roseus*);
- Aquifoliaceae (*Ilex paraguariensis*—yerba mate);
- Amaryllidaceae (*Clivia miniata*, *Lycoris radiata*, *Crinum americanum*);
- Berberidaceae (*Berberis aquifolium*, *B. vulgaris*, *B. aristata*);
- Buxaceae (*Buxus sinica*);
- Colchicaceae (*Colchicum autumnale*);
- Erythroxylaceae (*Erythroxylum coca*);
- Fabaceae (*Sophora flavescens*);
- Lauraceae (*Litsea glutinosa*, *Neolitsea konishii*);
- Loganiaceae (*Strychnos nux*-*vomica*);
- Malvaceae (*Theobroma cacao*);
- Melanthiaceae (*Veratrum californicum*);
- Monimiaceae (*Peumus boldus*);
- Nitrariaceae (*Peganum harmala*);
- Papaveraceae (*Chelidonium majus*, *Papaver somniferum*);
- Piperaceae (*Piper nigrum*, *P. longum*, *P. sarmentosum*);
- Ranunculaceae (*Coptis chinensis*, *Hydrastis canadensis*);
- Rubiaceae (*Coffea arabica*—Arabica coffee, *Uncaria tomentosa*);
- Rutaceae (*Evodia rutaecarpa*, *Zanthoxylum simulans*, *Z. ailanthoides*, *Z. stelligerum*, *Z. nitidum*);
- Sapindaceae (*Paullinia cupana*—guarana);
- Solanaceae (*Capsicum annuum*, *Lycium chinense*, *Solanum lycocarpum*, *S. nigrum*);
- Menispermaceae (*Stephania tetrandra*).

In the same vein, the following chemical groups of alkaloids have been studied in glioblastoma models in vitro and in vivo as single chemical compounds (*n* = 30): amide alkaloid (piperine = bioperine) [15,16], aporphine alkaloid (boldine) [28], benzophenanthridine alkaloids (chelerythine, dihydrochelerythine, nitidine) [29,30,31,32], beta-carboline alkaloids (harmine, harmaline) [33,34,35], bis-benzylisoquinoline alkaloid (tetrandrine) [36,37,38], glycoalkaloids (solamargine, α-solanine, solasonine) [17,39,40], indolopyridoquinazoline alkaloid (rutaecarpine) [41,42], isoquinoline alkaloid (berberine) [20,43,44,45,46,47,48,49,50], methylxanthine alkaloid (1,3,7-trimethylxanthine, caffeine) [51,52,53,54,55,56,57], non-narcotic opium alkaloid (papaverine) [58,59], oxindole alkaloids (pentacyclic and tetracyclic alkaloids) [60], phthalide-isoquinoline alkaloid (noscapine) [9], protoalkaloid (capsaicin) [61,62,63], pyrrolo-phenanthridine alkaloid (lycorine = narcissine) [64,65,66], quinazolinocarboline alkaloid (evodiamine) [41,67,68,69], quinolizidine alkaloid (oxymatrine) [70,71], spermine alkaloid (kukoamine A) [72], steroidal alkaloids (cyclopamine, cyclovirobuxine D) [73,74], tricyclic alkaloid (colchicine) [22,75,76,77], tropane alkaloid (cocaine) [78,79], and *Vinca* alkaloids (vinblastine and vincristine) [22,80,81,82].

### 1.3. The Use of Alkaloids in Potential Anticancer Therapies and Prognoses for Improving the Effectiveness of These Therapies

Recently, the number of scientific articles has increased, emphasizing the potential of alkaloids—a large group of plant secondary metabolites—to support oncological therapy. Alkaloids have been tested as anti-glioma drugs in glioblastoma (GBM) cell lines [83,84,85], as well as in retinoblastoma and neuroblastoma cells [86]. Some alkaloids, such as piperine and lycorine, have shown a synergistic effect with temozolomide in temozolomide-resistant human glioma cell lines by blocking the NF-κB pathway [87,88,89]. Additionally, alkaloids may act synergistically with radiation (e.g., piperine), increasing the radiation sensitivity of glioblastoma cells [90]. An increasing number of clinical studies are focusing on combining alkaloids (e.g., vinblastine) with carboplatin [91] or nilotinib [92], or using metronomic maintenance with vinblastine to prevent early relapse of pediatric low-grade glioma after treatment with bevacizumab and irinotecan [93]. Some alkaloids, such as evodiamine, are considered potential anticancer drugs [94].

However, many questions and doubts remain regarding the bioavailability of alkaloids, including their ability to penetrate the blood–brain barrier, pharmacokinetics, clinical efficacy, safety profile, and potential toxicity. These issues need to be investigated, explained, and discussed not only at the scientific level but also in practical oncological therapy. Consideration must be given to the different age groups and genders of glioblastoma patients and the various medications they take (including painkillers, anticancer drugs, and antiemetics). The systematic review shows that, among the large group of alkaloids, more than 30 alkaloids (including *Vinca* alkaloids: vinblastine and vincristine) have been studied over the past ten years. Available scientific publications (from the PubMed database) demonstrate that 13 alkaloids, including cyclopamine (*n* = 25), colchicine (*n* = 23), berberine (*n* = 17), caffeine (*n* = 14), capsaicin (*n* = 10), evodiamine (*n* = 8), piperine (*n* = 7), lycorine (*n* = 5), tetrandrine (*n* = 4), nitidine (*n* = 4), noscapine (*n* = 4), and alkaloids registered as chemotherapeutics (vincristine (*n* = 74) and vinblastine (*n* = 11)), are the most investigated in glioblastoma models.

Only studies focusing on the preclinical efficacy of single plant alkaloids, without combining them with other anticancer compounds (e.g., temozolomide), were selected for the original bibliographic analysis. This review summarizes the ten-year state of knowledge on the anti-glioma activity of alkaloids using in vitro, in vivo, and in silico methods. Similar research models employing glioblastoma cell lines and xenograft models in animals allow for the comparison of the activities, cellular and molecular mechanisms, and potential of different alkaloids.

In this current review, the focus is on bioavailability, which provides insight into the possible penetration of alkaloids through the blood–brain barrier. Attention is also given to new biopharmaceutical methods, including nanotechnology, which may find wider application in developing new and more effective anti-glioblastoma drugs based on plant alkaloids. Moreover, this review emphasizes not only the preclinical studies of alkaloids in pharmacological models of glioblastoma but also highlights the safety profile of alkaloids with cytotoxic activity. For this reason, the presented review stands out from others that focus primarily on biological activity and interactions with anticancer drugs.

This systematic review includes articles retrieved from the PubMed, Scopus, Web of Science, and Medline Complete databases, as well as The Cochrane Library, published from 2015 to 2024. To effectively search for publications, the following keywords were used: “glioblastoma and alkaloid”, “glioblastoma and natural therapy”, or the name of a single alkaloid combined with “glioblastoma.” Predatory journals listed on the website https://predatoryjournals.org/the-list (accessed on 31 January 2025) were excluded. The number of publications identified per year is as follows: 2024 = 26, 2023 = 21, 2022 = 32, 2021 = 39, 2020 = 28, 2019 = 29, 2018 = 41, 2017 = 29, 2016 = 42, and 2015 = 46, resulting in a total of 307 collected publications.

This review includes in vitro, in vivo, ex vivo, and in silico studies and review articles. For the original bibliographic analysis, only studies focusing on the preclinical efficacy of single plant alkaloids, without combining them with other anticancer compounds (e.g., temozolomide), were selected. Synergistic interactions between alkaloids (and other groups of phytocompounds) and temozolomide are discussed in another review [13].

In this review, 22 botanical families containing the most popular plant species with alkaloids are included. In summary, the anti-glioblastoma activity of 28 plant alkaloids was demonstrated and compared.

## 2. Current Status in Medicinal Products Based on Plant Alkaloids as Anticancer Drugs in Various Groups of Cancers

In the last 30 years, the FDA has approved only four drugs for the treatment of glioblastoma (GBM), including lomustine (e.g., Gleostine™), carmustine (e.g., BiCNU^®^), temozolomide (Temodal), and bevacizumab [95]. Lomustine, carmustine, and temozolomide are classified as simple alkylating agents (antineoplastic agents) with partial penetration to the brain. In contrast, bevacizumab is a monoclonal antibody that binds to vascular endothelial growth factor (VEGF) and prevents the activation of VEGF receptors on capillary endothelial cells [95]. However, these drugs have numerous side effects. Lomustine and carmustine can cause pulmonary toxicity (e.g., pulmonary fibrosis), hematologic toxicity (e.g., myelosuppression, thrombocytopenia, leukopenia, and bone marrow dysplasia), gastrointestinal toxicity (e.g., nausea and vomiting), hepatotoxicity, nephrotoxicity, and others [96]. Long-term therapy with temozolomide may lead to adverse events such as thrombocytopenia, neutropenia, pancytopenia, anemia, nausea, vomiting, anorexia, and myelosuppression [97,98]. An analysis of real-world pharmacovigilance and randomized clinical trials of bevacizumab showed adverse reactions, including nasal septum perforation, necrotizing fasciitis, and hypertensive encephalopathy [99].

The use of plant chemical matrices containing biologically active compounds, such as alkaloids, is justified in the development of new, safer, more effective, and potentially selective anticancer drugs to address the global oncological crisis [100,101]. Plant chemical matrices can be considered a chemical space in plants; metabolites biosynthesized in these plants can serve as a source for new drugs or precursors that can be optimized for developing potential analogs by applying modern principles of drug development based on plant-derived natural products.

Currently, plant-based medicines are a major source of anticancer therapies [101,102]. Many authors have long indicated that alkaloids and their semisynthetic and synthetic derivatives are important sources of anticancer drugs [13,100,101,102,103,104,105]. However, other chemical groups of plant secondary metabolites, such as flavonoids, phenylpropanoids, lactones, taxanes, and epipodophyllotoxins, have also demonstrated interesting anticancer effects [102]. Plant alkaloids have garnered significant interest as natural sources and continue to play a promising role in future drug discovery [102,103,104,105,106].

**Figure 1:**
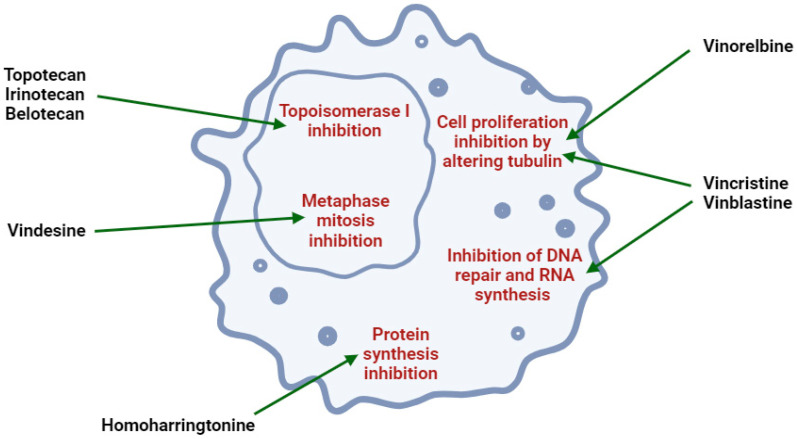
Mechanisms of pharmacological activities of the anticancer drugs based on plant alkaloids against cancer cells [figure presents one cancer cell and various targets to therapy]. Created using the BioRender.com.

Previous clinical studies have demonstrated the clinical efficacy and safety of many well-known plant alkaloids in oncological therapy. Several alkaloids have been registered by the US Food and Drug Administration (FDA) as pharmaceutical drug substances or chemotherapeutic agents for anticancer treatment (Figure 1, Table 1), including (1) vinblastine and vincristine: these *Vinca* alkaloids are natural alkaloids from the Madagascar periwinkle (*Catharanthus roseus*, Apocynaceae) [107,108]; (2) vindesine and vinorelbine: these are semi-synthetic derivatives of vinblastine extracted from *C. roseus* [107,108]; (3) topotecan, irinotecan, and belotecan: these are semi-synthetic derivatives of camptothecin (a quinoline alkaloid) extracted from the bark of the Chinese tree *Camptotheca acuminata* [84,109,110,111,112]; (4) homoharringtonine: this alkaloid is from *Cephalotaxus fortunei* (Taxaceae) [109,110,111,112]. In fact, only irinotecan and vincristine are considered for anti-glioblastoma therapy [111].

In addition to these alkaloids, other plant-derived chemical compounds recognized as pharmaceutical substances or chemotherapeutic drugs approved by the FDA include (1) Etoposide and Teniposide, which are semisynthetic derivatives of podophyllotoxin from the rhizome of *Podophyllum peltatum* [108,109,110,111,112,113,114,115] and (2) taxanes, which are diterpenes, mainly paclitaxel (Taxol), derived from the bark of the Pacific yew (*Taxus brevifolia*) [108,116].

On the other hand, according to updated safety information, camptothecin, vincristine, and vinblastine have exhibited severe toxic effects, including neurotoxicity and abdominal problems [85]. For this reason, continued research is warranted to develop new and safer anti-glioblastoma drugs, including other alkaloid substances.

## 3. Penetration/Transport of Selected Alkaloids Across the Blood–Brain Barrier—In Vitro and In Vivo Studies (Progress in Basic Research)

The human brain is an exceptionally complex organ that has developed an effective protection system to prevent the toxic effects of pathogens and neurotoxins. Consequently, designing new drugs capable of overcoming this protective system and achieving optimal concentrations at the desired therapeutic target in the brain is a significant challenge for medicinal chemists [117]. The blood–brain barrier (BBB) is a biological structure composed of microvascular endothelial cells, astrocytes, pericytes, and tight junctions, which collectively maintain the internal environment of the central nervous system (CNS) [118]. As a highly selective barrier, the BBB regulates the exchange of chemical substances between the bloodstream and the brain’s interstitial fluid, preventing the entry of potentially harmful compounds [119,120,121]. From a therapeutic perspective, the BBB presents a major obstacle for anticancer drugs with large molecular masses, as their ability to penetrate the barrier depends on several factors. These include the perfusion rate (e.g., cerebral blood flow and cerebrospinal fluid circulation), the paracellular and transcellular diffusion rate (determined by the drug’s physicochemical properties), and the concentration of the free (non-protein-bound) form of the anticancer drug in plasma [122]. The efficacy of drug transport into the brain is primarily influenced by transporters (mediating transporter-mediated transcytosis, mainly via P-glycoprotein), efflux pumps (energy-dependent mechanisms), and receptors (involved in receptor-mediated transcytosis) [122,123]. It is well known that anticancer compounds must possess dual molecular characteristics, appropriate solubility and molecular weight, to effectively cross the BBB [124,125]. Specifically, small lipid-soluble molecules can generally penetrate the BBB if their molecular mass is below 400 Da, and they form fewer than eight hydrogen bonds. Furthermore, the BBB effectively excludes approximately 100% of large-molecule compounds and over 98% of all small-molecule drugs [124,125]. One feasible approach to improving BBB permeability is increasing the lipophilicity of anticancer compounds. Currently, advanced drug delivery strategies, such as nanotechnology (nanomedicine), are being explored to enhance brain drug penetration [123,126]. However, in the case of alkaloids, the exact mechanisms and pathways influencing their BBB permeability remain incompletely understood. Since lipophilicity and molecular weight are key physicochemical properties affecting BBB penetration, each alkaloid should be studied individually to assess its potential for effective brain delivery.

In the search for new anticancer substances, including drugs for glioblastoma treatment, the blood–brain barrier (BBB) remains a major challenge in delivering chemotherapeutic agents to the brain [127]. According to Nooran and de la Rosa [121], improving the effectiveness of glioblastoma treatment requires enabling anticancer drugs to penetrate the BBB and reach the tumor directly to eliminate cancer cells.

The unsatisfactory efficiency of anti-glioblastoma therapy is not only due to the restrictive nature of the BBB but also to additional factors such as the blood–tumor barrier (BTB), the development of multidrug resistance (MDR) mediated by P-glycoprotein (P-gp, also known as MDR1 or ABCB1), and the formation of vasculogenic mimicry [127,128,129]. P-gp-mediated drug efflux is a key mechanism contributing to the poor bioavailability of xenobiotics [130,131]. Since P-gp, an efflux transporter protein, is expressed in cerebral microvascular endothelial cells in both the BBB and BTB, it presents a promising target for anticancer alkaloids [132,133,134]. Additionally, other natural compounds, such as resveratrol, have been explored as alternative P-gp inhibitors [135,136,137,138]. Currently, pharmacological approaches used in glioblastoma treatment have not yielded satisfactory results in clinical practice. According to Wang [120] remodeling the BBB represents a promising strategy for improving anti-glioblastoma therapy. Similarly, Wu [139] suggests that nanotechnology has emerged as a promising platform in this regard. Poorly bioavailable phytochemicals, including several alkaloids that cannot cross the BBB, should be encapsulated in nanoparticles to enhance absorption, transport, and stability [140]. Advanced nanoparticle delivery systems, such as liposomes, microspheres, and polymeric nanoparticles, should be further investigated to improve therapeutic efficiency [131]. According to recent studies, nanoformulations containing topotecan offer significant advantages in enhancing anticancer activity. Research indicates that topotecan-loaded nanocarrier systems exhibit superior pharmacokinetic properties, biocompatibility, tumor-targeting capabilities, and stability compared to native topotecan [141]. However, despite these emerging therapeutic strategies, they have not yet been widely adopted in clinical oncology practice [139].

To date, no systematic comparative studies using nanosystems have been conducted on alkaloids that inhibit glioma development at both the in vitro and in vivo levels. Furthermore, the correlation between the cytotoxic effects of alkaloids on glioblastoma cells and their ability to cross the blood–brain barrier (BBB) is not always observed within the same experimental timeframe. However, the permeability of several alkaloids through the BBB has been assessed using various experimental models beyond nanosystems and glioblastoma models, primarily among alkaloids containing nitrogen heterocycles.

**Figure 2:**
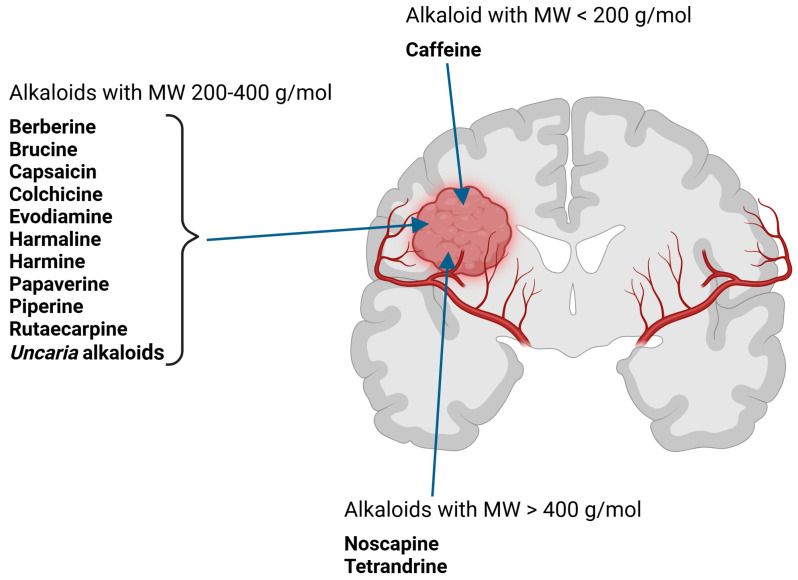
Predicted alkaloids crossing the blood brain-barrier after taking into account their molecular weight and the results of in vitro studies. Figure presents a scheme of a cross-section of a fragment of the blood–brain barrier with endothelial cells, astrocytes, and neuronal cells. There are two ways to cross the BBB: passive diffusion or active transport. Passive diffusion is a route preferred by most chemical compounds when provided they are lipid-soluble, have a molecular weight less than 650 g/mol and a logP value between 1.5 and 3.0, and are not too polar [156]. Created using the BioRender.com.

Several alkaloids have been studied for their transport across the BBB, including isoquinoline alkaloids: berberine, papaverine [58,59], noscapine [83,142,143], and tetrandrine [127,128,130,144]; indole derivatives: brucine [145], rutaecarpine [16,130], evodiamine [69,146], harmine [34,147], harmaline [148,149], vinblastine [150,151], and vincristine [127]; purine-like alkaloid: caffeine [51,152]; piperidine alkaloid: piperine [153,154]; and protoalkaloids: capsaicin and colchicine [127,150,151,155], The molecular weights (MWs) of the studied alkaloids, based on PubChem data, indicate their potential for BBB penetration (generally < 400 Da): caffeine (194.19 g/mol) < harmine (212.25 g/mol) < harmaline (214.26 g/mol) < piperine (285.34 g/mol) < rutaecarpine (287.3 g/mol) < evodiamine (303.4 g/mol) < capsaicin (305.4 g/mol) < berberine (336.4 g/mol) < papaverine (339.4 g/mol) < brucine (394.5 g/mol) < *Uncaria* alkaloids [hirsuteine (366.5 g/mol), hirsutine (368.5 g/mol), isocorynoxeine (382.5 g/mol), cnoxeine (382.5 g/mol), isorhynchophylline (384.5 g/mol)] < colchicine (399.4 g/mol) < cyclopamine (411.6 g/mol) < noscapine (413.4 g/mol) < tetrandrine (622.7 g/mol) < vinblastine (811 g/mol). All of these alkaloids satisfy the condition of having fewer than eight hydroxyl groups in their chemical structure, which is another key factor influencing BBB permeability (Figure 2).

It is important to highlight that the central nervous system multiparameter optimization (CNS MPO) desirability composite score was developed by Pfizer as an assessment tool during drug discovery to predict BBB permeability [157]. The CNS MPO score is obtained by summarizing individual components associated with six key physicochemical descriptors: molecular weight (MW), calculated logarithm of partition coefficient (ClogP), calculated logarithm of distribution coefficient at pH 7.4 (ClogD7.4), topological polar surface area (TPSA), number of hydrogen bond donors (HBDs), and the pKa of the most basic atom [158]. Additionally, several in silico BBB permeability prediction models are currently being developed and optimized [158].

Beyond computational methods, the importance of in vitro models has been increasingly recognized. These models can be classified into two-dimensional (2D) systems, such as monolayer and transwell models (both monoculture and co-culture), and three-dimensional (3D) models [159]. These in vitro systems should be employed to study alkaloid transport across the BBB more comprehensively while considering the limitations outlined by Ureña-Vacas [159]. In this context, piperine has already been investigated using these models [153,154]. Furthermore, the role of the BBB in limiting drug delivery and treatment efficacy remains a topic of discussion and ongoing controversy, particularly in the pathogenesis of high-grade brain tumors such as glioblastoma [95].

### 3.1. Caffeine

The results of the current meta-analysis indicate that higher coffee consumption is associated with a lower risk of glioma [160]. It is well known that the hydrophobic properties of caffeine enable it to cross biological membranes via simple diffusion [160]. Previous studies have demonstrated that caffeine can readily penetrate the BBB [135,136,137]. However, according to Lin [51], its precise effect on BBB permeability remains unclear, and its influence on glioblastoma cells has not been fully investigated [136]. Despite the well-established fact that caffeine crosses the BBB, key kinetic parameters governing its transport across the blood-cerebrospinal fluid barrier have not yet been determined [161], This gap in knowledge may be relevant for the potential use of caffeine in adjuvant glioma therapy. Similarly, theobromine, another methylxanthine alkaloid, has been shown to traverse the BBB in various experimental models [162,163,164,165]. Moreover, preliminary in vitro studies suggest that theobromine may exhibit anti-glioblastoma activity, including the inhibition of glioblastoma cell proliferation in culture [163].

### 3.2. Harmine and Harmaline

The indole alkaloid harmine has been shown to penetrate the BBB, suggesting its potential therapeutic effects in various brain disorders [166]. Additionally, harmine can rapidly enter the brain parenchyma following oral administration in mice [166]. In contrast, harmaline crosses the BBB, likely through the ATP-dependent efflux transporter BCRP (breast cancer resistance protein) [148]. Zetler et al. [149] reported that harmaline is taken up into the brain at a slower rate compared to harmine.

### 3.3. Piperine

Recent in silico studies indicate that piperine (1-piperoyl piperidine) can effectively cross the BBB [167]. Piperine and its analog SCT-64 demonstrated the highest BBB permeation potential via passive diffusion in various in vitro BBB models, including immortalized human BBB cells, human brain-like endothelial cells, and primary bovine endothelial/rat astrocyte co-cultures. In silico models further suggested that these alkaloids are unlikely to be substrates for active efflux transporters. Another study confirmed that piperine exhibits high BBB penetration potential without interactions with efflux transporters [168]. It showed a strong affinity for brain tissue (98.4–98.5%) and was detected in multiple brain regions, including the cortex, striatum, thalamus, hypothalamus, hippocampus, amygdala, cerebellum, midbrain, and brainstem. After oral administration in rats, piperine had a brain volume distribution of 36.32 ± 1.40 mL/g, with pharmacokinetic parameters of T(max) = 4 h, C(max) = 1395.53 ng/mL, and t1/2 = 2.39 h [145]. These findings suggest that piperine has strong brain tissue affinity and significant BBB penetration potential [168]. However, its precise transport mechanisms across the BBB remain unknown. Previously, piperine has been shown to enhance the bioavailability of various drugs, including diazepam, flunitrazepam, propranolol, warfarin, and salicylic acid [167,169]. Notably, piperine can synergistically enhance the anticancer effects of temozolomide against temozolomide-resistant glioma cell lines (U251MG, T98G) [87]. Additionally, it acts as a P-glycoprotein (P-gp) inhibitor [154].

### 3.4. Evodiamine and Rutaecarpine

Evodiamine has demonstrated the ability to penetrate the BBB [69]. Previously, Zhang et al. [130] observed that rutaecarpine also exhibits high BBB permeability. Using an MDCK-pHaMDR cell monolayer model, this alkaloid was shown to effectively cross the BBB [41].

### 3.5. Capsaicin

Capsaicin can cross the blood–brain barrier (BBB) [170,171]. Pharmacological studies have shown that, following intravenous administration in rats, capsaicin accumulates in the brain and spinal cord at concentrations five times higher than in the blood [172,173,174]. After subcutaneous administration, its distribution follows the order: brain > spinal cord > blood > skin [173]. According to Donnerer et al. [175], approximately 50% of capsaicin was detected in the rat brain within three minutes after an intravenous dose of 2 mg/kg and ninety minutes after a subcutaneous dose of 50 mg/kg.

Recent studies using nanotechnology have demonstrated that capsaicin, when formulated as nanoparticles with methoxy polyethylene glycol-poly(caprolactone) (mPEG-PCL), efficiently crosses the BBB. Human glioblastoma U251 cells cultured with fluorescein-loaded nanoparticles showed nanoparticle uptake via endocytosis [174]. Additionally, neurochemical studies suggest that dihydrocapsaicin may help reduce BBB disruption [176].

### 3.6. Berberine and Derivatives

Recently, Wang [177] observed that a glucose-coated berberine nanodrug enhanced the transport of berberine across the blood–brain tumor barrier in a mouse model. These results opened new possibilities for therapy since berberine cannot be administered orally and has low bioavailability when injected intravenously due to its insolubility and poor stability. In vitro studies using wild-type and P-gp-knockout mice showed that the substrates of P-gp included berberrubine, thalifendine, demethyleneberberine, jatrorrhizine, and columbamine, which are natural protoberberine alkaloids. An in vivo transport test using a Caco-2 monolayer demonstrated that the efflux capacity ranked as follows: berberrubine > berberine > columbamine ~ jatrorrhizine > thalifendine > demethyleneberberine. In silico studies also indicated that these alkaloids have an affinity for binding to P-gp [130].

### 3.7. Papaverine

Bhattacharjee et al. [178] revealed that intracarotid infusion of 0.1–0.2% papaverine in rats caused disruption of the blood–brain barrier (BBB) and increased the transport of sucrose into various brain regions. This effect was observed in a dose-dependent manner for the parietal cortex and brain stem. However, at a concentration of 0.15% papaverine, there was a significant increase in sucrose concentration in the frontal cortex, thalamus, hypothalamus, and contralateral fronto-parietal cortex.

### 3.8. Brucine and Strychnine

Brucine and strychnine, two major alkaloids of Bi Qi capsules (BQCs), possess the ability to penetrate the blood–brain barrier (BBB) effectively. Brucine acts as a substrate for P-glycoprotein (P-gp), whereas strychnine may function as an inhibitor of P-gp in the rat brain, as demonstrated using the microdialysis technique [145]. It was also observed that free strychnine and brucine were transported across the BBB into the brain. However, the high permeability of these alkaloids across the BBB was noted only at medium and high doses, which can induce neurotoxic effects (particularly for strychnine). Previous studies [179] have shown that P-gp participates in the transport process of brucine at the BBB, and it was observed that when brucine was used in conjunction with P-gp inhibitors, the concentration of brucine in the rat brain increased.

### 3.9. Uncaria Alkaloids

Alkaloids are the primary secondary metabolites found in the genus Uncaria, with over 100 alkaloids identified in these pantropical plants. Among the eight tested alkaloids from *Uncaria* (i.e., *Uncaria rhynchophylla* and *U. hirsuta*), hirsuteine and hirsutine (at 10 μM) significantly reduced the levels and function of P-glycoprotein (P-gp) in MCF-7/ADR cells. Furthermore, isocorynoxeine, corynoxeine, and isorhynchophylline suppressed P-gp mRNA levels in MCF-7/ADR cells [133]. In tests using the MDCK-pHaMDR cell monolayer model as an in vitro surrogate for the blood–brain barrier (BBB), isorhynchophylline, isocorynoxeine, hirsutine, and hirsuteine exhibited high permeability. These chemical compounds demonstrated time- and concentration-dependent effects and passive diffusion during their passage through the BBB [38].

### 3.10. Colchicine

The study revealed that P-glycoprotein (P-gp) limits the uptake of colchicine. Moreover, it was observed that vinblastine can inhibit its own transport across the blood–brain barrier in rats [150]. The results from other tests using the in situ rat brain perfusion technique showed that the volumes of distribution of colchicine and vinblastine did not differ among the eight gray matter areas of the brain. However, the distribution volumes of these alkaloids remained small. It is known that both colchicine and vinblastine are substrates of P-glycoprotein, but it can be assumed that P-gp is not the only barrier for these two chemical compounds [151].

### 3.11. Noscapine

Another isoquinoline alkaloid, noscapine, along with its analogs, demonstrated high penetration through the blood–brain barrier [9]. In an in vitro experimental model, noscapine crossed the simulated blood–brain barrier at a rate 31.8% more efficiently than morphine [143].

### 3.12. Tertrandrine (with Borneol, Vinorelbine, Vincristine)

Estimates of the safety, bioavailability, and pharmacokinetic parameters of tetrandrine are still very limited in animal models, mainly in clinical trials [131]. Recently, it was found that tetrandrine has the potential to improve drug penetration through the blood–brain barrier (BBB) and the blood-tumor barrier (BTB). Tetrandrine, in combination with borneol (a terpene derivative), reduced the integrity of the BBB in in vivo brain metastasis models. Moreover, tetrandrine (10 mg/kg/day), administered alone and with borneol (300 mg/kg/day) for five consecutive days to mice, inhibited the function of the P-gp efflux pump [128]. Another study showed that liposomes constructed with a tripeptide (Arg-Gly-Asp), tetrandrine, vinorelbine (an indole alkaloid anticancer drug), and DSPE-PEG2000 significantly enhanced transport across the BBB using glioma C6 cells, resistant C6 cells, and glioma-bearing mice [144]. Changes in pharmacokinetic parameters were identified, such as prolonged elimination half-life and increased AUC0-24h. It was observed that the liposomes acted through cellular and molecular mechanisms, including transporting/penetrating across the BBB, enhancing cellular uptake, downregulating P-gp, and inducing apoptosis via active substances in the liposomes. The following rank of inhibitory effects on both cancer cells after crossing the BBB was observed: RGD-modified vinorelbine plus tetrandrine liposomes > RGD-modified vinorelbine liposomes > vinorelbine plus tetrandrine liposomes > vinorelbine liposomes [144].

Furthermore, Song et al. [127] revealed that liposomes containing tetrandrine and vincristine (an indole alkaloid) modified with DSPE-PEG2000-NHS-transferrin (TF) accumulated at brain tumor sites in glioma-bearing mice due to improved physicochemical parameters for permeability and retention. This liposomal formulation exhibited the strongest cytotoxic effects on C6 cells and C6/ADR cells at various dose levels, with the following ranking of efficacy: liposomes with TF, tetrandrine, and vincristine > liposomes with tetrandrine and vincristine > liposomes with vincristine on C6 cells; and liposomes with TF, tetrandrine, and vincristine > liposomes with tetrandrine and vincristine > liposomes with TF and vincristine > liposomes with vincristine on C6/ADR cells. Moreover, liposomes with TF, tetrandrine, and vincristine crossed the BBB in vitro more effectively than other formulations. The activity of P-gp in various cell lines was reduced. Mice treated with liposomes containing TF, tetrandrine, and vincristine showed significantly longer survival times, along with blocking effects on cancer cell invasion and stronger induction of apoptosis in vitro via the upregulation of caspases [127].

## 4. Progress in Studies of Plant Alkaloids in Glioblastoma Models with Indication of Mechanism of Actions

A detailed analysis of the results showed that several mechanisms are similar for most of the alkaloids tested in a concentration-dependent manner, including the following effects observed in cell lines (in vitro model):

(1) Decreasing the viability of glioma cells;
(2) Suppressing cell proliferation;
(3) Inhibiting migration and invasion of glioma cells;
(4) Inducing apoptosis (increasing the percentage of apoptotic glioma cells);
(5) Decreasing the expression of Bcl-2 (an antiapoptotic marker) and other genes, as well as key signaling pathways.

Similarly, the following effects are observed in animal models (in vivo):

(1) Antiangiogenic effects;
(2) Decreasing tumor weight;
(3) Improving the survival rate of animals (Table 2).

Progress in biological and pharmacological research has been presented for groups of alkaloids with similar chemical structures (shown in Table 2).

### 4.1. Boldine, Berberine and Papaverine

Boldine (isoquinoline alkaloid; from aporphine alkaloid group) is widely distributed in several plants and is the main chemical compound biosynthesized in *Peumus boldus* (leaves and bark), the Chilean Boldo tree [180]. In addition to boldine, berberine and papaverine also belong to alkaloids with nitrogen heterocycles (true alkaloids). The current study showed that boldine treatment can reduce the proliferation of neural progenitor cells in the subventricular zone by inhibiting pannexin 1 hemichannels. Additionally, boldine can inhibit cell growth in all three tested GBM cell lines. It was observed that the U87-MG cell line was less sensitive to this alkaloid compared to the GBM59 and GBM96 cell lines [28]. Moreover, Noureini [181] revealed that boldine inhibits telomerase at sub-cytotoxic concentrations, suggesting that boldine may be a valuable candidate for telomerase-targeted cancer therapy. According to Pennisi et al. [182], future strategies may include anti-telomerase chemical compounds, which could lead to more effective anticancer treatments and improved outcomes for patients with glioblastoma. However, studies discussing this area remain limited.

Berberine (isoquinoline alkaloid; from protoperberine group) is commonly found in *Berberis vulgaris*, *Chelidonium majus*, and *Hydrastis canadensis* and is a popular alkaloid found in dietary supplements, potentially affecting immune system activity. In vitro studies have shown that berberine induces apoptosis in glioma cells by reducing Bcl-2 protein expression [46]. It also inhibits several signaling pathways, including TGF-β1/SMAD2/3 [50], and the phosphorylation of VEGFR2 and the ERK [44,47], while decreasing the AMPK/mTOR/ULK1 pathway. Additionally, berberine alters cell cycle progression, increasing the percentage of glioblastoma cells in the sub-G1 phase [45], and induces cell cycle arrest [47]. Another mechanism of action of berberine is the inhibition of glioma cell migration and invasion by suppressing the TGF-β1/COL11A1 pathway [183]. Moreover, berberine can inhibit glioblastoma cell proliferation in vitro by activating wild-type p53 or inhibiting mutant p53 activity [184].

Papaverine (isoquinoline alkaloid) is a non-narcotic opium alkaloid derived from *Papaver somniferum*, which has demonstrated anticancer activity in various in vitro models, including prostate carcinoma, colorectal carcinoma, breast carcinoma, fibrosarcoma, and hepatocarcinoma [142]. This alkaloid suppresses cancer cell migration (T98G cell line) and inhibits cell proliferation (U87MG and T98G cell lines) by inhibiting HMGB1 (high-mobility group box 1 protein, which is involved in cell migration and tumor metastasis) and RAGE (receptor for advanced glycation end products, which plays a role in tumor cell growth, migration, and invasion). Additionally, papaverine inhibits RAGE-dependent nuclear factor-κB activation [142]. According to Inada et al. [58] these findings suggest that papaverine may be effective against human glioblastoma.

### 4.2. Chelerythrine, Dihydrochelerythrine and Nitidine

Chelerythrine belongs to derivatives of benzophenanthridine, similar to dihydrochelerythrine and nitidine. Chelerythrine has been found in *Chelidonium majus*, *Macleaya cordata*, *Sanguinaria canadensis*, and *Zanthoxylum asiaticum*. It exerts numerous biological and pharmacological effects, including antiviral, anti-inflammatory, anti-diabetic, antifungal, anti-parasitic, and anticancer activities [69,185]. The anticancer activity of chelerythrine has been demonstrated in various in vitro experiments involving leukemia, non-small cell lung cancer, triple-negative breast cancer, prostate cancer, liver cancer, and renal cancer [185]. Recently, it was shown that chelerythrine reduces the protein expression of p-ERK1/2 and p-Smad2/3 and inhibits the TGF-β1-ERK1/2/Smad2/3-Snail/ZEB1 signaling pathway, thereby decreasing glioblastoma progression in U251 and T98G cell lines [29]. SMAD2, SNAIL, and ZEB1 are transcription factors involved in TGF-β signaling and epithelial-to-mesenchymal transition, which enhance the invasive phenotype of GBM cells, promoting glioma cell invasion and migration [186]. Therefore, further research is needed to explore other aspects of chelerythrine’s role in glioblastoma biology. On the other hand, Wang et al. [32] found that chelerythrine can induce glioma cell death, and this effect is associated with RIP1/RIP3-dependent necroptosis rather than apoptotic cell death in glioma cells at an early stage. The authors emphasized that chelerythrine should be considered a novel therapeutic strategy for glioblastoma treatment.

Dihydrochelerythrine has been found in *Corydalis yanhusuo*, *Macleaya microcarpa*, *Bocconia arborea*, *Zanthoxylum simulans*, *Z. ailanthoides*, and *Z. stelligerum*. It is a well-known antifungal compound that activates the mitochondrial apoptotic pathway. A recent study conducted on various glioblastoma cell lines (U251, GL-15, C6) showed that dihydrochelerythrine increased the levels of NF-κB and β-catenin in the cytoplasmic fraction [30]. Moreover, this alkaloid significantly elevated IL-6 levels and upregulated the signal transducer and activator of transcription-3 (STAT3) in the U251 cell line. Fu et al. [187] highlighted that the IL-6/JAK/STAT3 signaling pathway plays a crucial role in the pathogenesis and progression of several malignancies, including glioblastoma. The inhibition of STAT3 has been proposed as a promising therapeutic approach for GBM patients, as previously described by Luwor et al. [188]. According to Wang et al. [178], STAT3 inhibition can promote glioblastoma cell apoptosis.

Nitidine, derived from the root of *Zanthoxylum nitidum* (Rutaceae), similarly to dihydrochelerythrine, inhibits the JAK2/STAT3 pathway. Through this mechanism, nitidine suppresses glioma cell proliferation, migration, and invasion while promoting glioma cell apoptosis [31].

### 4.3. Lycorine

Lycorine is a derivative of pyrrolo-phenanthridine (*Amarylis* alkaloids) and is found in *Clivia miniata*, *Lycoris radiata*, and *Crinum americanum*; it exhibits several biological activities, including antiviral, antibacterial, antimalarial, anti-inflammatory, and anticancer effects, including anti-glioblastoma properties [64,65,66,189]. Dong et al. [64] demonstrated that lycorine inhibits pyruvate dehydrogenase kinase-3 (PDK3) expression in vitro and in vivo, suppressing the growth of chemoresistant glioblastoma cells. PDK3 has been associated with poor prognosis and negative oncological outcomes in various cancer types [190,191]. As an oncogene in glioblastoma, PDK3 promotes glioblastoma cell progression [192]. Another study showed that lycorine blocks the phosphorylation of the epidermal growth factor receptor (EGFR) and reduces the mRNA expression levels of *EGF*, *EGFR*, *Bcl-xL*, and *Ki-67*, leading to alterations in the expression of cell survival, death regulators, and the metastasis-related MMP9 protein [65]. Moreover, lycorine upregulates the NF-κB inhibitor protein IκB; downregulates NF-κB phosphorylation protein p-p65 [66,88]; decreases *EGF*, *EGFR*, *Bcl-xL*, and *Ki-67* mRNA and protein levels [65]; and inhibits PDK3 expression [82]. Additionally, lycorine may induce apoptosis in glioblastoma cells through an EGFR-mediated mechanism. The authors of this study concluded that lycorine could be a promising candidate for glioblastoma therapy by inhibiting cell migration, proliferation, and colony formation. Lycorine may be considered a next-generation anticancer drug and could contribute to the development of novel biological strategies for treating various cancer types [193].

### 4.4. Noscapine

Noscapine (from phthalidisoquinoline group), an alkaloid derived from *Papaver somniferum*, selectively blocks well-established inflammatory transcription factors such as NF-κB (nuclear factor kappa-light-chain-enhancer of activated B cells), which plays a key role in the pathogenesis of several diseases, including Alzheimer’s disease, diabetes, colorectal cancer, and glioblastoma [83,183,194,195,196]. Numerous studies have shown that the NF-κB signaling pathway is a crucial factor in gene induction, promoting cell survival and proliferation [197]. Altinoz et al. [9] summarized that noscapine, by modulating the NF-κB signaling pathway, can reduce tumor cell survival, proliferation, invasion, and angiogenesis. Additionally, noscapine binds to β-tubulin at a site distinct from that of taxanes, colchicine, and vincristine (a drug used in the treatment of recurrent high-grade glioma) [142,198]. Compared to other antineoplastic agents, noscapine exhibits strong anti-inflammatory effects [83], adding another pharmacological advantage in anti-glioblastoma treatment. Given its promising molecular mechanisms, noscapine should be further investigated as a novel therapeutic candidate for glioblastoma.

### 4.5. Tetrandrine

Tetrandrine (a derivative of bis-benzylisoquinoline alkaloids), an alkaloid isolated from *Stephania tetrandra*, a traditional Chinese medicinal plant, has demonstrated anti-tumor activity against various cancers, including breast, liver, pancreatic, leukemia, lung, prostate, gastric, and colorectal cancer [36]. In an in vitro glioblastoma model, tetrandrine reduces the protein levels of c-FLIP, MCL-1, and XIAP [36]; inhibits key metastasis-related proteins (*p*-EGFR(Tyr1068), SOS-1, GRB2, Ras, *p*-AKT(Ser473), *p*-AKT(Thr308), NF-κB-p65, Snail, E-cadherin, N-cadherin, NF-κB, MMP-2, and MMP-9) [44]; and suppresses the neural stem cell properties of glioblastoma stem-like cells (GSLCs) by upregulating GSK3β and β-catenin [38]. Furthermore, tetrandrine exhibits pro-apoptotic activity in various glioblastoma cell lines (U87, U251, and GBM 8401/luc2) by increasing the active forms of caspase-3, -8, and -9 [36,38], upregulating Bax, inducing PARP cleavage, and downregulating Bcl-2 [38]. Cell death induced by this alkaloid has been shown to reduce tumor volume and size in GBM 8401/luc2-cell xenografted animals [36]. In animal models, tetrandrine effectively suppresses tumor growth and glioma angiogenesis in rats [199]. In GBM 8401-cell xenografted mice, tetrandrine decreases the expression of c-FLIP, MCL-1, and XIAP in tumor tissues [36]. Another key molecular mechanism of tetrandrine is its ability to reduce the nuclear translocation and expression of β-catenin, a marker of proliferating endothelial cells in glioblastoma [38,200].

### 4.6. Brucine, Uncaria Alkaloids, Tetradium Alkaloids, Rutaecarpine, Derivatives of β-Carboline

Brucine belongs to monoterpenoid indole alkaloids and it is a characteristic alkaloid found in the seeds of *Strychnos nux-vomica* (Loganiaceae), a tree native to Sri Lanka, India, and Australia. This tree is also a natural source of the highly toxic alkaloid strychnine [201]. Brucine has been shown to upregulate activating transcription factor 3 (ATF3), leading to apoptosis in glioblastoma cell lines [42]. It was observed that increased BAX expression resulted in a reduced survival rate of glioma cells (U251) and inhibited tumor growth in a xenograft animal model. Additionally, Liu et al. [41] demonstrated that brucine can induce ferroptosis by increasing iron levels, H_2_O_2_ production, and lipid peroxidation in vivo.

To date, more than 200 compounds have been isolated from plants of the *Uncaria* genus. Several *Uncaria* alkaloids from the indole group have shown anticancer and neuroprotective effects [133]. While pentacyclic oxindole alkaloids are known to promote apoptosis, the precise mechanisms underlying their anti-glioblastoma activity remain largely unknown.

Evodiamine is a quinazolinocarboline alkaloid belonging to indole alkaloids, derived from the fruits of *Evodia rutaecarpa*, and promotes apoptosis in glioblastoma cells in a concentration-dependent manner. It induces chromatin condensation, nuclear fragmentation, and apoptotic body formation by suppressing the PI3K/AKT pathway and activating the MAPK pathway (Table 3) [67]. Current research indicates that the PI3K/AKT pathway is frequently dysregulated in various cancers, playing a key role in cell proliferation, migration, apoptosis, and differentiation [67]. The main mechanism underlying the anti-glioblastoma activity of evodiamine has been described in cell lines such as U251 and LN229 (the latter isolated from the right frontal parieto-occipital cortex of a glioblastoma patient).

Rutaecarpine, another alkaloid from *Evodia rutaecarpa*, has been shown to inhibit U87 glioblastoma cell migration by activating the aryl hydrocarbon receptor (AhR) signaling pathway. Researchers concluded that rutaecarpine may be considered a potent AhR activator, leading to the suppression of glioblastoma cell migration [147].

Harmine and harmaline were isolated from the seeds of *Peganum harmala*. Recent studies have demonstrated that harmine exhibits promising anti-glioblastoma effects through various mechanisms, including the inhibition of glioblastoma cell proliferation and migration, as well as the blockade of EGF-mediated phosphorylation of FAK/AKT. Additionally, harmine has been shown to inhibit apoptosis in glioblastoma cells [33,34]. Similarly, harmaline also exerts anti-glioblastoma effects by suppressing the proliferation and migration of U87 cells. It induces apoptotic cell death by triggering sub-G1 cell cycle arrest and upregulating cell cycle-related genes, including *p21*, *p53*, and the pro-apoptotic *Bax* (at 208 µM). Furthermore, harmaline decreases the expression of MMP-2 and MMP-9, which are associated with tumor invasion and metastasis [35].

### 4.7. Piperine

Piperine (from the piperidine alkaloid group) has been isolated from *Piper nigrum*, *P. longum* (fruits), and *P. sarmentosum* (roots) of the *Piperaceae* family. These plants are among the most widely used species worldwide [86]. Piperine exhibits various pharmacological activities, including antioxidant, antimicrobial, anti-inflammatory, and antidepressant effects, and anticancer potential in colorectal, breast, promyelocytic leukemia, prostate, rectal, lung, and ovarian cancer cells [86,87]. Interestingly, a *Piper nigrum* extract devoid of piperine but contains other alkaloids also demonstrated anti-proliferative effects in vitro against breast, colorectal, lung, and neuroblastoma cancer cell lines [86]. A recent study showed that piperine increases the radiation sensitivity of glioblastoma cells, mainly through synergistic effects in combination with radiation. Additionally, piperine inhibited the growth of human glioblastoma T98G cells at concentrations ranging from 25 µM to 200 µM [90]. Jeong et al. [87] observed that piperine enhances the effect of temozolomide against temozolomide-resistant human glioma cell lines. Piperine has also been reported to increase apoptosis (both apoptotic and necrotic cells) and reduce colony-forming potential [89]. Senrung et al. [202] demonstrated that piperine can suppress neoangiogenesis induced by malignant glioma cells (U87) by interfering with the vascular endothelial growth factor (VEGF) signaling pathway, leading to a reduction in *VEGFR-2* transcript levels and a decrease in VEGF-A expression. Another study tested a nanogel formulation loaded with curcumin and piperine against U-251 MG glioblastoma cells [203]. This formulation was found to penetrate cells via endocytic pathways and induce caspase-3-related apoptosis in glioblastoma cells.

### 4.8. Colchicine

Colchicine belongs to protoalkaloids (alkaloids with nitrogen in the side chain), which has been isolated from *Colchicum autumnale* and *Gloriosa superba* and has long been used as a medicinal product for the treatment of gout [75]. It is well known that colchicine, as an antimitotic drug, disrupts the cellular cytoskeleton by inhibiting microtubule polymerization in glioma cells [22,34,75,76]. Currently, microtubules are considered critical targets for microtubule-targeting agents (MTAs), which are clinically used to eliminate various cancer cells. These include vinca alkaloids (vincristine and vinblastine) and taxanes [76,77]. Microtubule-targeting agents have shown promising activity against glioblastoma cells, including colchicine-site binder molecules, which are generally smaller than other natural compounds such as vinca alkaloids and taxanes [77]. According to Zottel [76], the involvement of the cytoskeleton in key cellular processes makes it an attractive therapeutic target for glioblastoma multiforme. Other researchers [204] have also highlighted microtubules as an important and beneficial research target for developing new chemotherapeutic agents, particularly potent tubulin inhibitors. Moreover, Xia [205] classified ten categories of tubulin inhibitors, including colchicine derivatives, indole hybrids, podophyllotoxin derivatives, and lignans. Colchicine has been shown to inhibit cell division and proliferation. It has previously been revealed to exert cytotoxic activity against colon and breast cancer [75].

**Figure 3:**
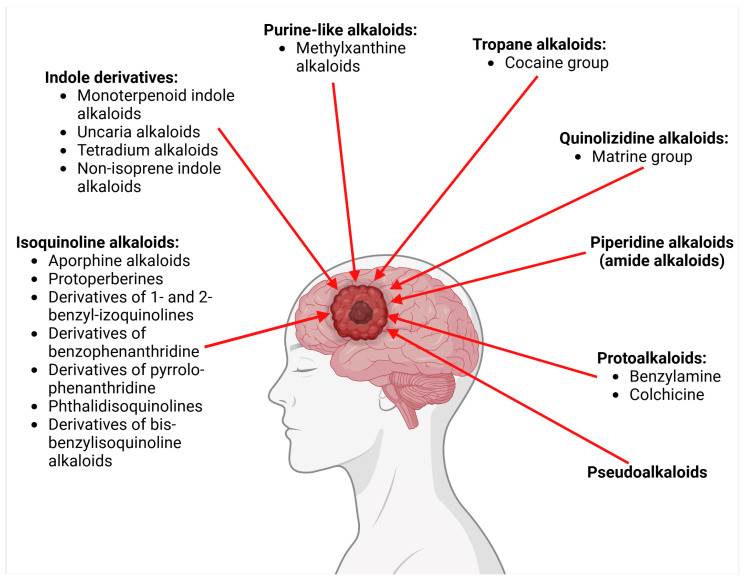
The most popular groups of plant alkaloids tested in glioblastoma models during the last ten years (2015–2024). Created using the BioRender.com.

The most popular groups of plant alkaloids tested in glioblastoma models are presented in Figure 3 and Table 2.

**Table 2:** Plant alkaloids tested in glioblastoma models during the last ten years (2015–2024).

**I. Alkaloids with nitrogen heterocycles (true alkaloids)**					
**1. Class: Isoquinoline alkaloids** **1.1. Major group: Aporphine alkaloids**					
Family name: Lauraceae, Monimiaceae					
No	Name of alkaloid	Natural source/derivative	Pharmacological model	Effect/IC_50_	Ref
1	Boldine	*Peumus boldus* (Monimiaceae), *Litsea glutinosa* (Lauraceae), *Neolitsea konishii* (Lauraceae)	glioma cell lines (GBM59, GBM96, U87-MG)	Inhibiting the hemichannel activity in neural progenitor cells—NPCs (at 50 μM), Reducing the proliferation of neural progenitor cells (at 50 μM) Decreasing the cell growth in a concentration-dependent manner (25–600 μM): IC_50_ = 68.6 μM (for GBM59 cells) IC_50_ = 141.7 μM (for GBM96 cells) IC_50_ = 213.8 μM (for U87-MG cells)	[28]
Chemical structure: 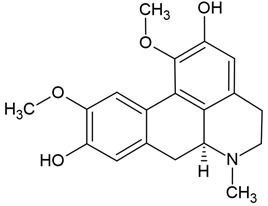				C_19_H_21_NO_4_ MW = 327.4 g/mol IUPAC Name: (6*aS*)-1,10-dimethoxy-6-methyl-5,6,6*a*,7-tetrahydro-4*H*-dibenzo[de,g]quinoline-2,9-diol	
**1.2. Major group: Protoperberines**					
Family name: Berberidaceae, Ranunculaceae					
2	Berberine - isoquinoline alkaloid	*Hydrastis canadensis* (Ranunculaceae), *Coptis chinensis* (Ranunculaceae), *Berberis aquifolium*, *Berberis vulgaris*, *Berberis aristata* (Berberidaceae)	glioma cell lines (U-87 and LN229)	Decreasing the viability of the U-87 cells and LN229 cells in a dose-dependent manner (from 1 μM to 40 μM), Obvious cytotoxicity (at 40 μM) Inhibiting migration and invasion of glioma cells in a dose-dependent manner (5 μM, 10 μM, 20 μM) Increasing the percentage of apoptotic glioma cells: 12.2% (at 5 μM), 20.56% (at 10 μM), 31.14% (at 20 µM) Inducing the apoptosis by downregulating Bcl-2, and upregulating Bax and caspase-3 Inhibiting the TGF-β1/SMAD2/3 signaling pathway in a dose-dependent manner (5 μM, 10 μM, 20 μM)	[43]
	Berberine		glioma cell lines (U87MG)	Suppressing the cell proliferation Increasing early apoptosis (53.5% ± 11.15) after application of berberine at concentration of 25 µM (24 h exposure) Reduction in the cell viability of U87MG cells in a concentration- and time-dependent manner	[20]
	Berberine		ectopic and orthotopic xenograft models in BALB/c nude mice	Anti-tumor and antiangiogenic effects of berberine (50 mg/kg by oral gavage for 28 days) Decreasing the tumor weight (50 mg/kg by oral gavage for 28 days) Improving the survival rate of mice (50 mg/kg by oral gavage for 28 days) Inhibiting the phosphorylation of VEGFR2 and ERK	[44]
	Berberine		(1) glioblastoma cell lines (U87 and U251) (2) the ectopic tumor xenograft mouse model	In vitro Inhibiting the cell viability of human glioblastoma cell lines U87 and U251 (IC_50_ of 42 and 32 μmol/L, respectively) Inhibiting the proliferation of U87 and U251 cells Inhibiting the cell migration of HUVEC by 67.50 ± 8.14% In vivo Reducing the tumor weight and improving the survival rate of mice (50 mg/kg berberine) Inhibiting the angiogenesis in glioblastoma xenografts	[44]
	Berberine		glioma cell lines (U87 and U251)	In vitro IC_50_ = 42 μM (line U87), IC_50_ = 32 μM (line U251)	[46]
	Berberine		glioma cell line (U343)	Reduction in the viability in U343 cells at concentration of 10 μM or 50 μM of berberin (60% and 29% viability, respectively) Altering cell cycle progression: 10 μM of berberine increased the percentage of U343 cells found in subG1 Inducing the autophagosome formation in U343 cells at 50 μM berberine	
	Berberine		glioma cell lines (U87 and U251)	Inhibition of the AMPK/mTOR/ULK1 pathway (50–200 μM) Attenuated cell proliferation in both U251 and U87 cells in a dose-dependent manner (50–200 μM) Inducing the apoptosis (50–200 μM) Regulating the Bax, Cytochrome C, cleaved caspase-3 Reducing the expression of Bcl-2 proteins Impairing the migration and invasion in cells (50–200 μM) Decreasing the oxidative phosphorylation in berberin-treated U251 and U87 cells Reducing the glycolytic capacity in cells (50–200 μM) Inducing the autophagy in cells (50–200 μM) Reducing the tumor growth (at concentration of 100 μM)	[46]
	Berberine	Berberine chloride	(1) glioma cell lines (U-87 MG, U251 MG, U-118 MG, and SHG-44) (2) U87 cells inoculated into the right striatum of mouse brains - berberine (50 and 100 mg/kg body weight) daily for 5 weeks.	In vitro: Reducing the cell viability in a dose-dependent and time-dependent manner IC_50_ = 21.76 µmol/L for 72 h (cell line U87) IC_50_ = 9.79 µmol/L for 72 h (cell line U251) IC_50_ = 35.54 µmol/L for 72 h (cell line U118) Inhibiting cell proliferation and induces cell cycle arrest in glioma cells (U87, U251) at 15–150 µmol/L of berberin Inhibition of the EGFR–RAF–MEK–ERK signaling pathway Increasing the percentage of senescent GBM cells: more than 70% (in U87 cells) and 40% (in U251cells) after 15 mmol/L of berberine (7 days) In vivo: Reduction in the tumor volume in mice Inhibition of the proliferation of glioma in mice Diminishing the level of EGFR	[47]
Chemical structure: 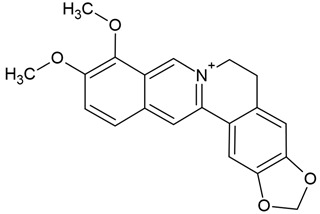				C_20_H_18_NO_4_^+^ MW = 336.4 g/mol IUPAC Name: 16,17-dimethoxy-5,7-dioxa-13-azoniapentacyclo[11.8.0.0^2,10^.0^4,8^.0^15,20^]henicosa-1(13),2,4(8),9,14,16,18,20-octaene	
**1.3. Major group: Derivatives of 1- and 2-benzyl-izoquinolines**					
Family name: Papaveraceae					
3	Papaverine (non-narcotic opium alkaloid)	*Papaver somniferum*	(1) human GBM U87MG, T98G cells (2) U87MG xenograft mouse model	Inhibiting the cancer cell proliferation: EC_50_ = 29 μM (U87MG cells) EC_50_ = 40 μM (T98G cells) Suppressing the tumor cell growth in a U87MG xenograft mouse model and reducing the tumor volume by 63% with papaverine treatment in comparison with the vehicle control (on day 47)	[59]
Chemical structure: 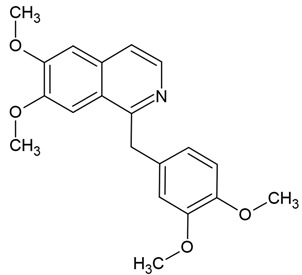				C_20_H_21_NO_4_ MW = 339.4 g/mol IUPAC Name: 1-[(3,4-dimethoxyphenyl)methyl]-6,7-dimethoxyisoquinoline	
**1.4. Major group: Derivatives of benzophenanthridine**					
Family name: Papaveraceae					
4	Chelerythrine	*Chelidonium maius*	glioblastoma cell lines (U251 and T98G); BALB/c nude mice	Inhibited the proliferation, stemness, migration, and invasion of glioblastoma cells, Inducing apoptosis Restraining the progression of xenograft tumors Decreasing the expression of Bcl-2 (an antiapoptotic marker) (2–8 µM of chelerythine) Increasing the expression of Bax (a pro-apoptotic marker) in glioblastoma cells (2–8 µM of chelerythine) Reducing the protein expression of p-ERK1/2 and p-Smad2/3 in a dose-dependent manner	[29]
	Chelerythrine		(1) glioma cell lines (rat C6 and human U87), (2) U87 xenograft animal model	A dose- and time-dependent reduction in cell viability cell lines, preferentially in U87, and C6 glioma cells: IC_50_ = 8–15 μM Inhibiting the colony formation and cell proliferation Inducing the RIP1/RIP3-mediated necroptosis Inducing the mitochondrial fission Initiating the process of mitophagy in C6 and U87 cells inhibiting the tumor growth via necroptosis in U87 mouse xenograft model	[32]
Chemical structure: 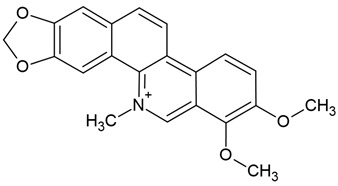				C_21_H_18_NO_4_^+^ MW = 348.4 g/mol IUPAC Name: 1,2-dimethoxy-12-methyl-[1,3]benzodioxolo[5,6-c]phenanthridin-12-ium	
Family name: Rutaceae					
5	Dihydrochelerythrine (DHC)	*Zanthoxylum simulans*, *Z. ailanthoides*, *Z. stelligerum*	human glioblastoma cells (U251, GL-15), murine glioblastoma cells (C6)	Decreasing the cell viability (C6 and U251) in a time- and dose-dependent manner (100 and 200 µM) Decreasing cell viability GL-15 after treatment of DHC at concentration of 200 µM Cytostatic effect after 100 µM DHC (after 48 h) in C6 and U251 cells Increasing level of the IL-6 in U251 cells Increasing level of NFκ B and β-catenin cytoplasmic fraction (C6 cells) STAT3 upregulation in U251 cells	[30]
Chemical structure: 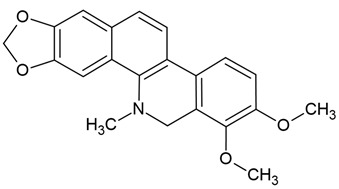				C_21_H_19_NO_4_ MW = 349.4 g/mol IUPAC Name: 1,2-dimethoxy-12-methyl-13*H*-[1,3]benzodioxolo[5,6-c]phenanthridine	
6	Nitidine chloride	*Zanthoxylum nitidum* (root)	human glioblastoma cell lines U87 and LN18	Inhibiting the proliferation of glioma cells: IC_50_ concentration for nitidine was between 5.0 and 7.5 μM (48 h) Inhibiting the migration and invasion abilities in glioma cells (in a concentration-dependent manner) Upregulation of the expression of epithelial marker E- cadherin at concentrations > 7.5 μM Promoting the glioma cell apoptosis in a concentration-dependent manner (2.5, 5.0, 7.5, and 10.0 μM) for 48 h Inhibiting the JAK2/STAT3 pathway Inhibiting the enhanced expression of stem cell markers	[31]
Chemical structure: 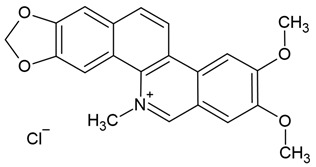				C_21_H_18_NO_4_^+^ MW = 348.4 g/mol IUPAC Name: 2,3-dimethoxy-12-methyl-[1,3]benzodioxolo[5,6-c]phenanthridin-12-ium;chloride	
**1.5. Major group: Derivatives of pyrrolo-phenanthridine (Amarylis alkaloids)**					
Family name: Amaryllidaceae					
7	Lycorine (narcissine)	*Clivia miniata*, *Lycoris radiata*, *Crinum americanum*	(1) glioblastoma cell line (U-87); (2) the protein–protein interaction (PPI) network (the STRING online database)	In vitro: IC_50_ = 3.68 μM at 48 h (in U-87 MG cells) IC_50_ = 2.85 μM (in C6 cells) [Su 2023] Inducing the apoptosis in U-87 MG cells The apoptosis rates: 17.24% (in 1.25 μM), 18.46% (in 2.5 μM), 21.28% (in 5 μM), and 22.98% (in 10 μM) Upregulating the NF-κB inhibitor protein IκB, the downregulation of the NF-κB phosphorylation protein p-p65 [Su 2023] Molecular docking results: Obtaining the 136 predicted targets of lycorine and GBM Strong binding efficiency of lycorine with the 10 key genes	[66,88]
	Lycorine (narcissine)		(1) molecular docking modeling assay (2) 10 cell lines (i.e., U87, LN229, U251, A172, Gli36vIII, GBM6) (3) In vitro EGFR kinase assay (4) xenograft models	Binding to the intracellular EGFR (696-1022) domain as an inhibitor of EGFR (IC_50_ was about 68 nM) IC_50_ = about 10 μM (U251 cells) Decreasing of GBM cellular proliferation (10–20 μM) Decreasing the cell migration (in a dose-dependent manner) Inhibiting the colony formation (at 10 μM) Inducing cell apoptosis in an EGFR-mediated manner Inhibiting the xenograft tumor growths in three animal models in vivo (at 20 mg/kg/day of lycorine completely blocked the growth of tumor in mice) At 25 μM fully blocked EGFR phosphorylation Decreasing the expression of mRNA of EGF, EGFR, Bcl-xL, Ki-67, and protein level	[65]
	Lycorine hydrochloride		(1) human GBM cells (temozolomide-resistant LN229 and U251 cells = 251R, 229R cells) (2) 229R xenograft mouse model	Inhibiting aerobic glycolysis, excessively activates mitochondrial respiration and promotes production of ROS (229R cells) IC_50_ = 2.17 μM (in 229R cells) IC_50_ = 2.39 μM (in 251R cells) Inhibiting the proliferation and invasive capacity of chemoresistant GBM cells (in dose-dependent manner, at 1 μM, 2 μM, and 4 μM) Inhibiting the chemoresistant GBM cell growth and lactate production in vivo (30 mg/kg/day) Inhibiting PDK3 expression in vitro and in vivo Supressing the aerobic glycolysis and oxidative phosphorylation hyperactivation by inhibiting PDK3	[64]
Chemical structure: 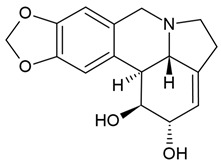				C_16_H_17_NO_4_ MW = 287.3 g/mol IUPAC Name: (1*S*,17*S*,18*S*,19*S*)-5,7-dioxa-12-azapentacyclo[10.6.1.0^2,10^.0^4,8^.0^15,19^]nonadeca-2,4(8),9,15-tetraene-17,18-diol	
**1.6. Major group: Phthalidisoquinolines**					
Family name: Papaveraceae					
8	Noscapine	*Papaver somniferum*	various glioblastoma cell lines	Suppressing the S-phase and colony growth of glioblastoma cells (noscapine at concentration ~ 11 μM), IC_50_ = 100 μM Suppressing the proliferation of rat C6 glioma cells Induced cell death in C6 glioma cells Reduction in tumor volume (~ 78%) after noscapine treatment (300 mg/kg, per os, daily) of mice with rat C6 glioblastoma implanted into their brain striatum Inhibiting the proliferation and inducing apoptosis of human glioma cell lines (IC_50_ from 85 to 131 μM) as a strong inhibitor	[83]
Chemical structure: 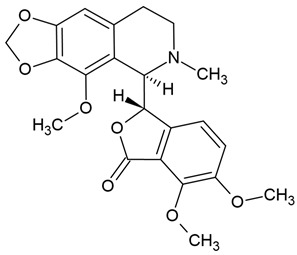				C_22_H_23_NO_7_ MW = 413.4 g/mol IUPAC Name: (3*S*)-6,7-dimethoxy-3-[(5*R*)-4-methoxy-6-methyl-7,8-dihydro-5*H*-[1,3]dioxolo[4,5-g]isoquinolin-5-yl]-3*H*-2-benzofuran-1-one	
**1.7. Major group: Derivatives of bis-benzylisoquinoline alkaloids**					
Family name: Menispermaceae					
9	Tetrandrine	*Stephania tetrandra* (root)	(1) GBM 8401/luc2 human glioblastoma cells (2) xenografted nude mice	In vitro: Decreasing the cell viability from 8.35 to 98.16% in a dose-dependent manner (at various concentrations: 10–25 μM) Inducing the apoptotic cell death from 23.81 to 74.59% in a dose-dependent manner (at 10–20 μM) In vivo: A two-fold decreasing the volumes of tumors at 50 mg/kg Reducing tumor weight at 25 and 50 mg/kg (on day 21) and higher dose showed more significant inhibition Decreasing the protein levels of c-FLIP, MCL-1, and XIAP (anti-apoptosis markers) Increasing the apoptosis-associated protein expressions (caspase-3, caspase-8, and caspase-9)	[36]
	Tetrandrine		GBM 8401 cells	Inhibiting the cell mobility, migration, and invasion of cells (1.5–10 μM) Inhibiting the key metastasis-related proteins (p-EGFR_(Tyr1068)_, SOS-1, GRB2, Ras, p-AKT_(Ser473)_, p-AKT_(Thr308)_, NF-κB-p65, Snail, E-cadherin, N-cadherin, NF-κB, MMP-2, and MMP-9) Reducing the MAPK signaling-associated proteins, reducing the NF-κB and DNA binding (in a dose-dependent manner)	[37,44]
	Tetrandrine		glioma stem-like cells (GSLCs) from the human glioblastoma cell lines U87 and U251	Inhibiting the cell viability of U87 GSLCs and U251 GSLCs: IC_50_ = 30.41 µM (in U87 GSLCs) IC_50_ = 27.5 µM (in U251 GSLCs) Inhibiting the neurosphere formation, the migration of U87 GSLCs and U251 GSLCs in a dose-dependent manner (0–10 µM) Inducing the apoptosis of GSLCs (by the upregulation of Bax, the cleavage of PARP, and the downregulation of Bcl-2) at concentrations of 10 and 20 µM Inhibiting the neural stem cell properties of GSLCs with the upregulation of GSK3β and β-catenin at 20 µM, Inhibiting the GSLCs by repressing the nuclear translocation and expression of β-catenin at 20 µM	[38]
Chemical structure: 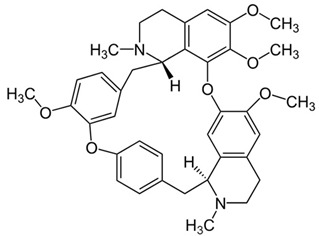				C_38_H_42_N_2_O_6_ MW = 622.7 g/mol IUPAC Name: (1*S*,14*S*)-9,20,21,25-tetramethoxy-15,30-dimethyl-7,23-dioxa-15,30-diazaheptacyclo[22.6.2.2^3,6^.1^8,12^.1^14,18^.0^27,31^.0^22,33^]hexatriaconta-3(36),4,6(35),8,10,12(34),18,20,22(33),24,26,31-dodecaene	
**2. Class: Indole derivatives**					
**2.1. Group: Monoterpenoid indole alkaloids**					
Family name: Loganiaceae					
No	Name of alkaloid	Natural source/derivative	Pharmacological model	Effect/IC_50_	Ref.
1	Brucine		glioblastoma lines (U118, U87, U251, and A172)	Inhibiting the viabilities of human U251, U87, U118, and A172 glioma cells in a dosage-dependent manner (from 50 to 800 µmol/L) Could obviously inhibit the U87 and U251 glioma cells to form colonies (12.5–25 μM brucine) Inducing the upregulation of the activating transcription factor 3 (ATF3) Improving the ferrous iron and lipid peroxidation in glioma cells in a dosage- and time-dependent manner of brucine (250–500 µM) Inducing the generation of malondialdehyde (MDA) Inducing the ferrous iron-dependent lipid peroxidation and glioma cell death	[42]
Chemical structure: 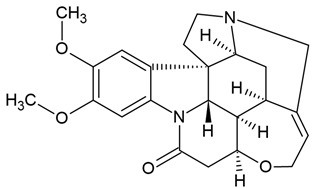				C_23_H_26_N_2_O_4_ MW = 394.5 g/mol IUPAC Name: (4*aR*,5*aS*,8*aR*,13*aS*,15*aS*,15*bR*)-10,11-dimethoxy-4*a*,5,5*a*,7,8,13*a*,15,15*a*,15*b*,16-decahydro-2*H*-4,6-methanoindolo[3,2,1-ij]oxepino[2,3,4-de]pyrrolo[2,3-h]quinolin-14-one	
**2.2. Group: Uncaria alkaloids**					
Family name: Rubiaceae					
2	Oxindole alkaloids-pentacyclic alkaloids	*Uncaria tomentosa* (stem bark and leaves)	human glioblastoma cell line-U-251-MG	Selectivity index (SI) against malignant cells: SI = 1.11–3.04 for chemotype II SI = 0.10–0.19 for chemotype I SI = 0.21–0.57 for chemotype III Hirsuteine (MW = 366.5 g/mol), hirsutine (MW = 368.5 g/mol), isocorynoxeine (MW = 382.5 g/mol), corynoxeine (MW = 382.5 g/mol), and isorhynchophylline (MW = 384.5 g/mol)	[60]
**2.3. Group: Tetradium alkaloids**					
Family name: Rutaceae					
3	Evodiamine (quinazolinocar-boline alkaloid)	*Evodia rutaecarpa* = *Tetradium ruticarpum*	human GBM cell lines U251 and LN229	Inhibited the cell proliferation in a time- and dose-dependent manner Increasing the percentages of early and late apoptotic cells (at 10 µM for 24 h): from 3.8% to 13.8% of early apoptotic cells (in U251 cells); from 2.5% to 20.5% (in LN229); from 2.9% to 16.2% (in U251); from 5.0% to 13.7% (LN229) Induced apoptosis in cancer cells by suppressing PI3K/AKT Signaling and inducing MAPK phosphorylation (p38 and JNK, but not ERK) to regulate apoptotic proteins (Bax, Bcl-2, Cytochrome c, Caspase-3, and PARP) Induced reactive oxygen species (ROS) production and mitochondrial membrane potential (MMP) disruption	[67]
Chemical structure: 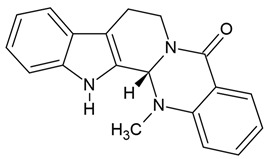				C_19_H_17_N_3_O MW = 303.4 g/mol IUPAC Name: (1*S*)-21-methyl-3,13,21-triazapentacyclo[11.8.0.0^2,10^.0^4,9^.0^15,20^]henicosa-2(10),4,6,8,15,17,19-heptaen-14-one	
4	Rutaecarpine (indolopyridoquinazoline alkaloids) (in comparison with other main alkaloids: evodiamine, dehydroarutaecar-pine)	*Evodia rutaecarpa*	U87 human glioblastoma cells	Decreasing the migration ability of U87 cells in an AhR-dependent manner; the inhibition rate of rutaecarpine (10^−5^ M) = 25.4% (24 h) The migration distance of cells after rutaecarpine (10^−5^ M) was decreased by 32.5% (at 12 h), 37.9% (at 24 h), and 39.5% (at 36 h) Inductive effect on CYP1B1 mRNA expression (in time and concentration-dependent manner) Potent aryl hydrocarbon receptor (AhR) activator (leading to inhibition of cell migration) Involving the AhR signaling pathway in mechanism of rutaecarpine activity	[41]
Chemical structure: 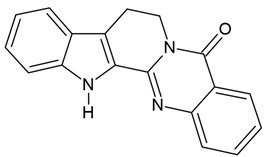				C_18_H_13_N_3_O MW = 287.3 g/mol IUPAC Name: 3,13,21-triazapentacyclo[11.8.0.0^2,10^.0^4,9^.0^15,20^]henicosa-1(21),2(10),4,6,8,15,17,19-octaen-14-one	
**2.4. Group: Non-isoprene indole alkaloids—Derivatives of β-carboline**					
Family name: Nitrariaceae					
5	Harmine	*Peganum harmala* (the seeds)	glioblastoma (GBM) cell lines (U251-MG and U373-MG cells)	Suppressing the proliferation of cells (anti-proliferative effect) in a dose-dependent way (5–50 µM) through EGF-mediated FAK/AKT pathway Inhibiting the cell viability (5–50 µM) Restraining the cell survival of glioblastoma cells in a dose and time-dependent way (10, 20, 30 µM) for 24, 48, or 72 h Reduction in the phosphorylation of FAK and AKT in glioblastoma cells (10 µM of harmine) Inhibiting the migration and promotes apoptosis in GBM and affects the expression of related proteins Inhibiting the migration of U251-MG cells	[33,34]
Chemical structure: 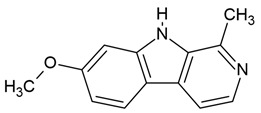				C_13_H_12_N_2_O MW = 212.3 g/mol IUPAC Name: 7-methoxy-1-methyl-9*H*-pyrido[3,4-b]indole	
6	Harmaline	*Peganum harmala* (the seeds)	human malignant glioblastoma cell line (U-87)	IC_50_ = 208 for 24 h IC_50_ = 127 µM for 48 h Suppressing the proliferation of U-87 cells Inducing sub-G1 cell cycle arrest and apoptotic cell death The percentage of late apoptotic = 75.1% (in 208 µM) The percentage of necrotic cells = 20.1% (in 208 µM) Upregulating the cell cycle-related genes: p21, p53, pro-apoptotic Bax (at 208 µM) Decreasing the expression of MMP-2 and MMP-9 (at 208 µM) Reducing the enzymatic activity of MMP-2 and MMP-9 (at 52 µM after 24 h) Inhibiting the migration of U-87 cells Inducing the production of ROS (at 208 µM)	[35]
Chemical structure: 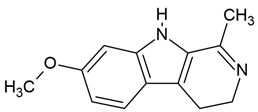				C_13_H_14_N_2_O MW = 214.3 g/mol IUPAC Name: 7-methoxy-1-methyl-4,9-dihydro-3*H*-pyrido[3,4-b]indole	
**3. Class: Purine-like alkaloids**					
**3.1. Group: Methylxanthine alkaloids**					
Family name: Aquifoliaceae, Malvaceae, Rubiaceae, Sapindaceae					
No	Name of alkaloid	Natural source/derivative	Pharmacological model	Effect/IC_50_	Ref.
1	Caffeine (1,3,7-trimethylxanthine)	*Coffea arabica* (Rubiaceae), *Paulinia cumana* (Sapindaceae), *Ilex paraguariensis* (Aquifoliaceae), *Theobroma cacao* (Malvaceae)	glioblastoma line: U87-MG (with or without temozolomid (500 μM TMZ)	A concentration below 1 mM did not affect the viability of cells (U87-MG) Inducing the cell death through mitotic catastrophe Abrogating TMZ-induced G2/M arrest through inhibiting ATM-p53-p21 pathway Enhancing the anti-proliferative effect after combination of caffeine (1 mM) and TMZ (500 μM) in comparison with TMZ alone (500 μM)	[51]
	Caffeine		glioblastoma cell lines (U-87MG and LN229)	Reducing the survival rate of GBM8401 cells to 70% after treatment of caffeine at concentration of 1 mM (for 24 h) Reducing the survival rates of U-87MG and LN229 cells by 30% after 5 mM caffeine (for 24 h) Reducing the invasion of glioma cells through ROCK-cathepsin B/FAK/ERK signaling pathway after treatment with caffeine (0.1 mM and 0.5 mM) for 24 h Decreasing the mRNA, protein expression, and activity of cathepsin B	[52]
	Caffeine		glioblastoma lines (C6 and U87MG)	Inhibiting the proliferation of glioma cells Inducing the apoptosis of glioma cells Treatment for 24 h with caffeine significantly blocked the cell cycle in the G0/G1 phase Reduction in the expression of protein Bcl-2 Inhibition of the cell viability in dose-dependent manner (1–20 mM of caffeine) Maximal non-cytotoxic concentration of caffeine on both glioblastoma cell lines was 0.5 mM	[53]
Chemical structure: 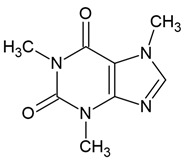				C_8_H_10_N_4_O_2_ MW = 194.2 g/mol IUPAC Name: 1,3,7-trimethylpurine-2,6-dione	
**4. Class: Tropane alkaloid**					
**4.1. Group:** **Cocaine group**					
Family name: Erythroxylaceae					
No	Name of alkaloid/class of alkaloids	Natural source/derivative	Pharmacological model	Effect/IC_50_	Ref.
1	Cocaine	*Erythroxylum coca*	C6 glioblastoma cells	IC_50_ = 6.76 mM after 24 h No difference between the IC 50 after 24 and 72 h App. 53% of the cells in apoptosis and 21% in necrosis after 15 mM of cocaine	[79]
Chemical structure: 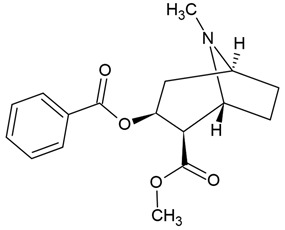				C_17_H_21_NO_4_ MW = 303.4 g/mol IUPAC Name: methyl (1*R*,2*R*,3*S*,5*S*)-3-benzoyloxy-8-methyl-8-azabicyclo[3.2.1]octane-2-carboxylate	
**5. Class: Quinolizidine alkaloids**					
**5.1. Group: Matrine group**					
Family name: Fabaceae					
No	Name of alkaloid/class of alkaloids	Natural source/derivative	Pharmacological model	Effect/IC_50_	Ref.
1	Oxymatrine	*Sophora flavescens*	U251MG human malignant glioma cells	Reduction in the cell viability in a dose- and time-dependent manner (at various concentrations: 0.25-4 mg/mL) Reducing the protein expression levels of cyclin D1, CDK4, and CDK6 Inducing the apoptosis (at concentrations: 0.5, 1, and 2 mg/mL) Suppressing the invasion of glioblastoma cells (at various concentrations: 0.25 mg/mL, 0.5 mg/mL, 1 mg/mL, and 2 mg/mL) Inhibition of glioma cell proliferation	[70]
Chemical structure: 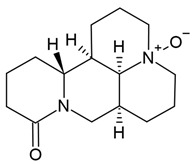				C_15_H_24_N_2_O_2_ MW = 264.4 g/mol IUPAC Name: (1*R*,2*R*,9*S*,17*S*)-13-oxido-7-aza-13-azoniatetracyclo[7.7.1.0^2,7^.0^13,17^]heptadecan-6-one	
**6. Class: Piperidine alkaloids (amide alkaloids)**					
Family name: Piperaceae					
No	Name of alkaloid/class of alkaloids	Natural source/derivative	Pharmacological model	Effect/IC_50_	Ref.
1	Piperine (bioperine; 1-piperoylpiperidine)	*Piper nigrum*, *P. longum (fruits)*, *P. sarmentosum* (roots)	human cells T98G	Inhibiting the growth of the cells (25 µM, 50 µM, 75 µM, 100 µM 150 µM, and 200 µM) IC_50_ = 133 µM (after a six days incubation period) Piperine showed synergistic effects in combination with radiation Demonstrating the radiation-sensitizing effect of piperine in higher concentrations Manifold genotoxic effects of piperine	[90]
	Piperine (bioperine; 1-piperoylpiperidine)		(1) human GBM U87 cells; (2) GBM cancer stem cells (GSCs); (3) in silico; the cancer genome atlas (TCGA) database	IC_50_ = 120 µM (48 h) 75% reduction in survivin expression in the S cells 50% reduction in survivin expression in the P cells Piperine exerted the potential as a survivin inhibitor (GBM and GSCs) Inducing apoptosis (apoptotic and necrotic cells) Reduction in colony-forming potential (GSCs)	[89]
Chemical structure: 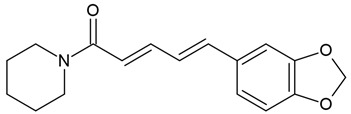				C_17_H_19_NO_3_ MW = 285.3 g/mol IUPAC Name: (2*E*,4*E*)-5-(1,3-benzodioxol-5-yl)-1-piperidin-1-ylpenta-2,4-dien-1-one	
**II. Protoalkaloids–alkaloids with nitrogen in the side chain**					
**1. Class: Benzylamine**					
Family name: Solanaceae					
No	Name of alkaloid/class of alkaloids	Natural source/derivative	Pharmacological model	Effect/IC_50_	Ref.
1	Capsaicin (N-vanillyl-8-methyl-alpha-nonenamide) -a lipophilic protoalkaloid	Capsicum genus i.e., *Capsicum annuum*	glioblastoma cell line (LN-18)	Decreasing the viability of glioblastoma cells through TRPV1-independent mechanism: IC_50_ = 325.7 ± 12.4 μM at 24 h of treatment IC_50_ = 265.7 ± 10.2 μM at 48 h of treatment Stimulating apoptosis Inducing the expression of PPARγ in glioblastoma LN-18 cells by capsaicin in a dose-dependent manner Decreasing collagen biosynthesis in glioblastoma cells Potentiating the cytotoxicity of thiazolidinediones	[61]
	Capsaicin		glioblastoma cell lines (U87-MG and U251)	Concentration-dependent anti-proliferative effects Inducing redox imbalance Inducing ferroptosis through ACSL4/GPx4 signaling pathways	[62]
	Capsaicin and methoxy polyethylene glycol-poly(caprolactone) (mPEG-PCL) in nanoparticles		human glioblastoma cells (U251)	Inhibiting the growth of cells in a concentration- and time-dependent manner by capsaicin-loading nanoparticles and capsaicin Higher cytotoxicity of capsaicin-loading nanoparticles in comparison with capsaicin: - Inhibitory rates of capsaicin = 43 ±3.8% - Inhibitory rates of capsaicin-loading nanoparticle = 68 ± 2.9%	[175]
Chemical structure: 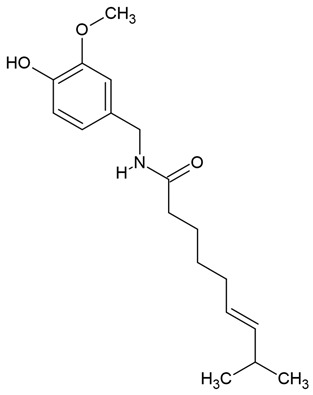				C_18_H_27_NO_3_ MW = 305.4 g/mol IUPAC Name: (*E*)-*N*-[(4-hydroxy-3-methoxyphenyl)methyl]-8-methylnon-6-enamide	
**2. Class: Colchicine**					
Family name: Colchicaceae					
2	Colchicine derivative (tricyclic alkaloid)	*Colchicum autumnale*	(1) glioblkastoma cell lines (U87MG and U373MG) (2) rat glioma animal model	IC_50_ = 10 nM induced a 50% reduction in the cell viability of U87MG cells IC_50_ = 50 nM induced a 50% reduction in the cell viability of U373MG cells Changing in cellular cytoskeleton by inhibition of polymerization of the microtubule Increasing formation of autophagosomes and autophagy vesicles Reduction in tumor size by about 80% after injection of lipid-based formulated colchicine derivative (0.5 mg/kg, 2.5 mg/kg) in rats	[75]
Chemical structure: 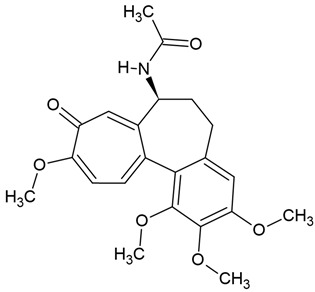				C_22_H_25_NO_6_ MW = 399.4 g/mol IUPAC Name: *N*-[(7*S*)-1,2,3,10-tetramethoxy-9-oxo-6,7-dihydro-5*H*-benzo[a]heptalen-7-yl]acetamide	
**III. Pseudoalkaloids**					
**1. Class: steroidal alkaloids**					
Family name: Solanaceae					
No	Name of alkaloid/class of alkaloids	Natural source/derivative	Pharmacological model	Effect/IC_50_	Ref.
1	α-Solanine (glycoalkaloid)	*Solanum nigrum* and *Solanum tuberosum*, and *Solanum aculeastrum*	(1) glioma cells (2) Traditional Chinese Medicine Systems Pharmacology Database (3) GeneCards, networks (STRING online database)	Association of α-solanine with several signaling pathways with positive regulation of MAP kinase activity and PI3K-Akt Inhibiting the proliferation and migration by α-solanine (10 µM and 15 µM) Promoting the apoptosis of glioma cells α-solanine as a potential mediator STAT1	[39]
Chemical structure: 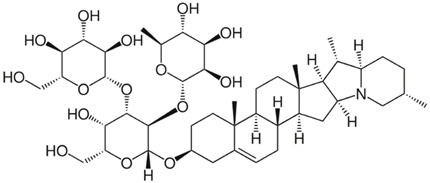				C_45_H_73_NO_16_ MW = 868.1 g/mol IUPAC Name: 2-[5-hydroxy-6-(hydroxymethyl)-2-[(10,14,16,20-tetramethyl-22-azahexacyclo[12.10.0.0^2,11^.0^5,10^.0^15,23^.0^17,22^]tetracos-4-en-7-yl)oxy]-4-[3,4,5-trihydroxy-6-(hydroxymethyl)oxan-2-yl]oxyoxan-3-yl]oxy-6-methyloxane-3,4,5-triol	
Family name: Melanthiaceae					
1	Cyclopamine (steroidal alkaloid), cyclopamine glucuronide prodrug		in vitro, ex vivo, and in vivo: - glioma stem cells (GSCs) - C6 rat GBM cells	Reduction in the tumor density IC_50_ = 11.4 μM and 7.7 μM for C6 cells and C6-GSCs, respectively (in the presence of β-glucuronidase) Reduction in the colony formation in a time-dependent manner after prodrug 1b with β-glucuronidase and cyclopamine (at 10 μM) Max. reduction in colonies grown of about 50% (prodrug with β-glucuronidase) and 80% (cyclopamine) after 72 h of treatment Increasing the caspase-3/7 by 50 and 80% in C6 cells and C6-GSCs after treatment of 1b+β-glucuronidase, and by 100 and 130% with cyclopamine	[73]
Chemical structure: 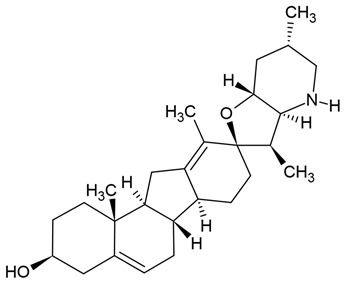				C_27_H_41_NO_2_ MW = 411.6 g/mol IUPAC Name: (3*S*,3′*R*,3′*aS*,6′*S*,6*aS*,6*bS*,7′*aR*,9*R*,11*aS*,11*bR*)-3′,6′,10,11*b*-tetramethylspiro[2,3,4,6,6*a*,6*b*,7,8,11,11*a*-decahydro-1*H*-benzo[a]fluorene-9,2′-3*a*,4,5,6,7,7*a*-hexahydro-3*H*-furo[3,2-b]pyridine]-3-ol	
Family name: Buxaceae					
5	Cyclovirobuxine D (CVBD)	*Buxus sinica* (Buxaceae)	glioblastoma (GBM) cell lines (T98G, U251)	Anti-proliferation effect in dose- and time-dependent manners (80, 120, 160 µM) Inducing the apoptosis, and mitochondrial damage in GBM cells Inducing the mitochondrial translocation of cofilin, Activation of cleaved- caspase3 Degradation of PARP with formation of cleaved PARP Inducing the autophagy associated with the AKT/mTOR pathway	[73]
Chemical structure: 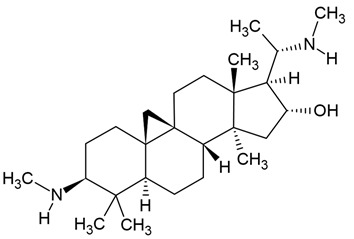				C_26_H_46_N_2_O MW = 402.7 g/mol IUPAC Name: (1*S*,3*R*,6*S*,8*R*,11*S*,12*S*,14*R*,15*S*,16*R*)-7,7,12,16-tetramethyl-6-(methylamino)-15-[(1*S*)-1-(methylamino)ethyl]pentacyclo[9.7.0.0^1,3^.0^3,8^.0^12,16^]octadecan-14-ol	
**IV. Polyamines alkaloids**					
	Kukoamine A	*Lycium chinense*, potatoes, and tomatoes	human GBM cells (U251and WJ1)	Decreasing the proliferation, colony formation, migration, and invasion of GBM cells Arresting G0/G1 phase in the cell cycle Decreasing the growth of tumors Decreasing the 5-Lipoxygenase and antiapoptotic protein Bcl-2 expression Increasing the apoptotic cells Increasing the apoptotic proteins, Bax, and caspase-3 expression	[72]
Chemical structure: 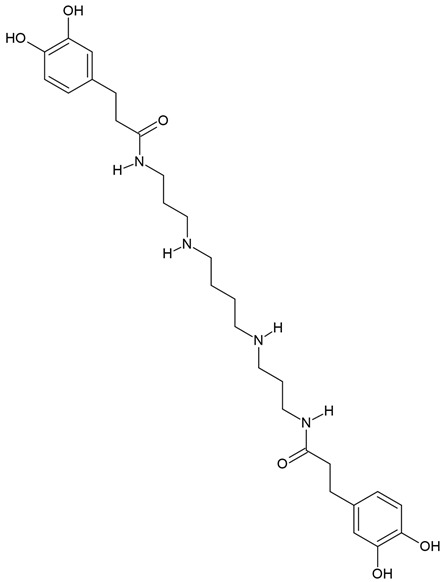				C_28_H_42_N_4_O_6_ MW = 530.7 g/mol IUPAC Name: 3-(3,4-dihydroxyphenyl)-*N*-[3-[4-[3-[3-(3,4-dihydroxyphenyl)propanoylamino]propylamino]butylamino]propyl]propanamide	

## 5. Comparison of Cytotoxicity and Safety Profile of Selected Alkaloids

Toxicology plays a crucial role throughout the drug discovery and development process [206]. A proper scientific approach to developing new anti-glioblastoma drugs requires a thorough evaluation of potential toxicity and an assessment of the benefit–risk ratio for patient groups [207]. This screening process can help exclude certain alkaloids as drug candidates for further development. Additionally, artificial intelligence may play a promising role in predicting toxicity and optimizing drug safety [208]. A review of bibliographic data from the past ten years allows for the classification of alkaloids into three categories:

1. High-toxicity alkaloids (where risks outweigh benefits);
2. Limited-toxicity alkaloids (where benefits exceed risks);
3. Alkaloids under investigation in liposomal and nanoformulations (to mitigate toxicity concerns).

Among the alkaloids with greater therapeutic benefits than risks and potential as novel drug candidates, the following have been identified: noscapine (exerting negligible toxicity and non-addictive activity), lycorine (low toxicity), capsaicin (nanoparticles with capsaicin may reduce adverse effects and improve efficacy), chelerythrine (liposomal formulations modified with polyethylene glycol can reduce toxicity and enhance its anti-glioblastoma activity), caffeine (inhibiting proliferation and reducing the invasion of glioma cells), boldine (good safety profile but low cytotoxic activity against glioblastoma cells), and colchicine (a microtubule-targeting agent but with incomplete eradication of glioblastoma cells).

Furthermore, this systematic review revealed that, despite their observed cytotoxic activity against glioblastoma cells, some alkaloids may pose greater risks than potential therapeutic benefits. From this perspective, an analysis of toxicological data on tetrandrine has shown that this alkaloid can cause liver and lung damage in animals, along with several toxic side effects in humans; therefore, its use as an anti-glioblastoma drug can be excluded. Similarly, nitidine has demonstrated toxicity in the liver, kidneys, and heart, along with cardiotoxic effects in animal models. Harmine and harmaline exhibit hallucinogenic effects and interfere with serotonin activity in the brain. Cyclopamine has shown teratogenic potential in animals. Cocaine exerts toxic effects on multiple organs and has addictive properties. Additionally, the serious toxicity of brucine may limit its clinical applications.

Piperine remains controversial due to its toxic effects on the reproductive system and its association with hemorrhagic ulceration in the gastrointestinal tract. Moreover, due to unfavorable pharmacokinetic parameters (i.e., poor bioavailability), novel pharmaceutical formulations using nanotechnology are needed for berberine.

**Figure 4:**
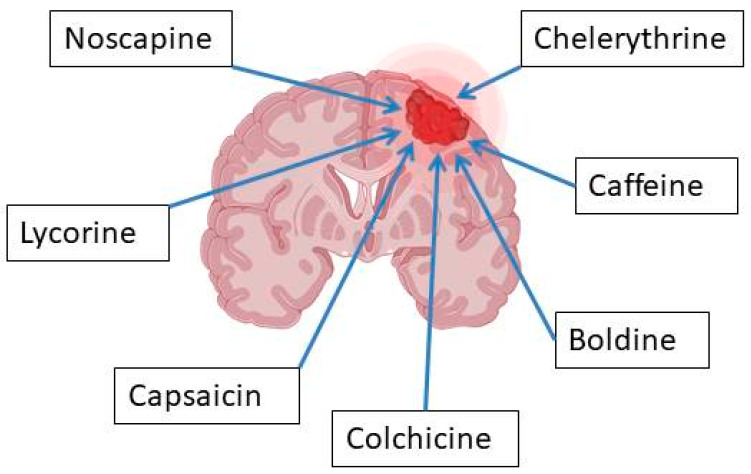
Selected alkaloids considered as new drug candidates based on a review that allows for the consideration of greater pharmacological benefits than observed toxicity and side effects. Created using the BioRender.com.

For several alkaloids, the validity of further clinical research in drug development cannot yet be assessed due to the limited availability of pharmacological and toxicological data. Taken together, kukoamine A, cyclovirobuxine D, α-solanine, oxymatrine, rutaecarpine, and evodiamine should be further investigated in the context of toxicity evaluation. A summary of the detailed toxicological profiles of selected alkaloids, along with an assessment of the need for further studies, is presented in Table 3. Selected alkaloids considered as new drug candidates are shown in Figure 3 and Figure 4.

## 6. Conclusions

New compounds with potential anti-glioblastoma activity should meet the following criteria: appropriate physicochemical properties (lipophilicity, molecular mass < 400 Da, <8 hydrogen bonds) and optimized pharmacokinetic and pharmacodynamic profiles. These include selective cytotoxicity against glioblastoma cells, blood–brain barrier (BBB) permeability, P-glycoprotein efflux liability, dose optimization, and maximum tolerated dose assessment. Additionally, safety, toxicity, adverse drug reactions, and potential drug–drug interactions must be evaluated during preclinical and clinical development [232]. Preclinical studies face methodological inconsistencies, making comparisons difficult. Standardized protocols are needed, especially in cell line studies, incorporating functional assays such as morphological analysis, proliferation assays (MTT), apoptosis assays, and migration studies. Cytotoxicity should also be evaluated alongside standard glioblastoma treatments under both normoxic and hypoxic conditions.

There is a lack of systematic in silico studies, including computational assessments (e.g., CNS MPO score), and comparative research on BBB and blood–cancer barrier penetration. A comprehensive understanding of alkaloid pharmacokinetics (oral bioavailability, distribution, metabolism, and elimination) remains limited. Current BBB transport models may not directly reflect glioblastoma patient outcomes, necessitating further research into pharmacokinetics–pharmacodynamics correlations. Additionally, alkaloid toxicity on neural tissue, particularly glial cells, requires systematic evaluation.

Several alkaloids show potential as glioblastoma treatments: noscapine, lycorine, capsaicin, chelerythrine, caffeine, boldine, and colchicine. However, some alkaloids, despite cytotoxic activity, pose high risks, including tetrandrine, nitidine, harmine, harmaline, cyclopamine, cocaine, and brucine. Piperine remains controversial due to reproductive toxicity and gastrointestinal hemorrhagic ulceration. Additionally, berberine’s poor bioavailability necessitates nanotechnology-based formulations. Further investigation is needed for kukoamine A, cyclovirobuxine D, α-solanine, oxymatrine, rutaecarpine, and evodiamine.

This review highlights significant progress in alkaloid research for glioblastoma treatment, yet major gaps remain, particularly in BBB penetration studies and targeted drug delivery systems. More comprehensive preclinical and translational research is essential to advance these compounds toward clinical applications.

## Figures and Tables

**Table 1:** Medicinal products based on plant alkaloids as anticancer drugs.

Name of Anticancer Drug	Natural Alkaloid as Natural Matrices for Drug/Medicinal Plants	Mechanism of Action	Recommendations/Registered Clinical Trials	Ref.
Topotecan	Semi-synthetic derivative of camptothecin (quinoline alkaloid) extracted from the bark of the tree *Camptotheca acuminata* (Chinese tree)	Topoisomerase I inhibitors, apoptosis	Ovarian cancer (FDA May 1996) Cervical cancer (FDA June 2006) Small cell lung carcinoma (FDA October 2007) Experimental uses: for glioblastoma	[112]
Chemical structure 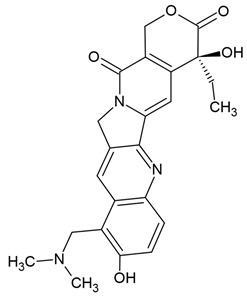			C_23_H_23_N_3_O_5_ MW = 421.4 g/mol IUPAC name: (19*S*)-8-[(dimethylamino)methyl]-19-ethyl-7,19-dihydroxy-17-oxa-3,13-diazapentacyclo[11.8.0.0^2,11^.0^4,9^.0^15,20^]henicosa-1(21),2,4(9),5,7,10,15(20)-heptaene-14,18-dione	
Irinotecan	Semi-synthetic derivative of camptothecin (quinoline alkaloid) extracted from the bark of the tree *Camptotheca acuminata* (Chinese tree)	Topoisomerase I inhibitors	Colon cancer Small cell lung cancer (with cisplatin) Metastatic pancreatic adenocarcinoma (FDA Feb. 2024) [Nalirifox -irinotecan liposome, in combination with oxaliplatin, fluorouracil, and leucovorin] Glioblastoma (in combination with other anticancer drug: bevacizumab)	[109,111,113]
Chemical structure 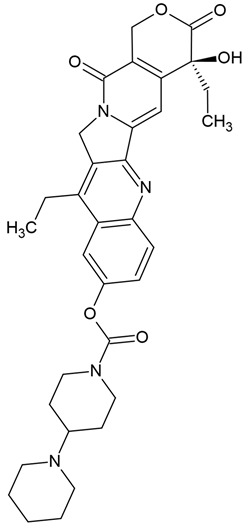			C_33_H_38_N_4_O_6_ MW = 586.7 g/mol IUPAC name: [(19*S*)-10,19-diethyl-19-hydroxy-14,18-dioxo-17-oxa-3,13-diazapentacyclo[11.8.0.0^2,11^.0^4,9^.0^15,20^]henicosa-1(21),2,4(9),5,7,10,15(20)-heptaen-7-yl] 4-piperidin-1-ylpiperidine-1-carboxylate	
Belotecan	Semi-synthetic derivative of camptothecin (quinoline alkaloid) extracted from the bark of the tree *Camptotheca acuminata* (Chinese tree)	Topoisomerase I inhibitors	Small cell lung carcinoma Ovarian cancer	[110]
Chemical structure 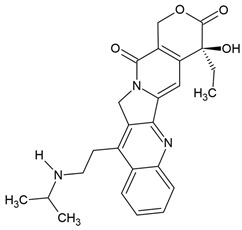			C_25_H_27_N_3_O_4_ MW = 433.5 g/mol IUPAC name: (19*S*)-19-ethyl-19-hydroxy-10-[2-(propan-2-ylamino)ethyl]-17-oxa-3,13-diazapentacyclo[11.8.0.0^2,11^.0^4,9^.0^15,20^]henicosa-1(21),2,4,6,8,10,15(20)-heptaene-14,18-dione	
Vincristine	Natural alkaloid from the Madagascar periwinkle *Catharanthus roseus*	Increasing in cancer cell death Cell proliferation inhibition by altering tubulin Binding to intracellular tubulin Inhibiting DNA repair and RNA synthesis by inhibiting DNA-dependent RNA polymerase enzyme	Non-Hodgkin’s lymphoma Lymphoblastic leukemia Neuroblastoma Wilkins’s tumor Hodgkin’s disease	[107]
Chemical structure 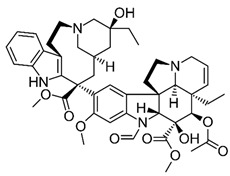			C_46_H_56_N_4_O_10_ MW = 825.0 g/mol IUPAC name: methyl (1*R*,9*R*,10*S*,11*R*,12*R*,19*R*)-11-acetyloxy-12-ethyl-4-[(13*S*,15*S*,17*S*)-17-ethyl-17-hydroxy-13-methoxycarbonyl-1,11-diazatetracyclo[13.3.1.0^4,12^.0^5,10^]nonadeca-4(12),5,7,9-tetraen-13-yl]-8-formyl-10-hydroxy-5-methoxy-8,16-diazapentacyclo[10.6.1.0^1,9^.0^2,7^.0^16,19^]nonadeca-2,4,6,13-tetraene-10-carboxylate	
Vinblastine	Natural alkaloid from the Madagascar periwinkle *Catharanthus roseu*	Increasing in cancer cell death Cell proliferation inhibition by altering tubulin Binding to intracellular tubulin Inhibiting DNA repair and RNA synthesis by inhibiting DNA-dependent RNA polymerase enzyme	Breast cancer Lung cancer Head and neck cancer Hodgki’s lymphoma Testicular cancer	[91,92,93,107]
Chemical structure 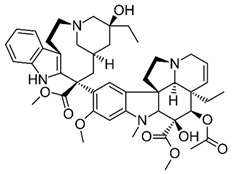			C_46_H_58_N_4_O_9_ MW = 811.0 g/mol IUPAC name: methyl (1*R*,9*R*,10*S*,11*R*,12*R*,19*R*)-11-acetyloxy-12-ethyl-4-[(13*S*,15*R*,17*S*)-17-ethyl-17-hydroxy-13-methoxycarbonyl-1,11-diazatetracyclo[13.3.1.0^4,12^.0^5,10^]nonadeca-4(12),5,7,9-tetraen-13-yl]-10-hydroxy-5-methoxy-8-methyl-8,16-diazapentacyclo[10.6.1.0^1,9^.0^2,7^.0^16,19^]nonadeca-2,4,6,13-tetraene-10-carboxylate	
Vindesine	Semi-synthetic derivatives of vinblastine extracted from *Catharanthus roseus*	Preventing cells from entering metaphase mitosis	Leukemia Non-small cell lung cancer Lymphoma	[107]
Chemical structure 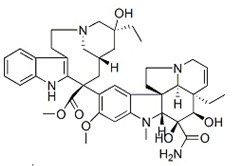			C_43_H_55_N_5_O_7_ MW = 753.9 g/mol IUPAC name: methyl (13*S*,15*S*,17*S*)-13-[(1*R*,9*R*,10*S*,11*R*,12*R*,19*R*)-10-carbamoyl-12-ethyl-10,11-dihydroxy-5-methoxy-8-methyl-8,16-diazapentacyclo[10.6.1.0^1,9^.0^2,7^.0^16,19^]nonadeca-2,4,6,13-tetraen-4-yl]-17-ethyl-17-hydroxy-1,11-diazatetracyclo[13.3.1.0^4,12^.0^5,10^]nonadeca-4(12),5,7,9-tetraene-13-carboxylate	
Vinorelbine	Semi-synthetic derivatives of vinblastine extracted from *Catharanthus roseus*	Suppresses cell proliferation by attaching to tubulin	Breast cancer Non-small cell lung cancer	[107]
Chemical structure 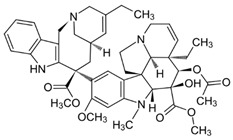			C_45_H_54_N_4_O_8_ MW = 778.9 g/mol IUPAC name: methyl (1*R*,9*R*,10*S*,11*R*,12*R*,19*R*)-11-acetyloxy-12-ethyl-4-[(12*S*,14*R*)-16-ethyl-12-methoxycarbonyl-1,10-diazatetracyclo[12.3.1.0^3,11^.0^4,9^]octadeca-3(11),4,6,8,15-pentaen-12-yl]-10-hydroxy-5-methoxy-8-methyl-8,16-diazapentacyclo[10.6.1.0^1,9^.0^2,7^.0^16,19^]nonadeca-2,4,6,13-tetraene-10-carboxylate	
Homoharringtonine	*Cephalotaxus fortunei*	A protein synthesis inhibitor	Severe myeloid leukemia (approved by the FDA)	[111]
			Hematologic malignancies solid tumors: Phase I; NCT01844869 Hematologic tumors: Phase I; NCT00675350 Leukemia: Phase III; NCT00004933 Acute myelogenous leukemia: Phase II; NCT01873495 Leukemia: NCT02159872; Phase II Chronic myeloid leukemia: Phase I/II; NCT02078960	[102]
Chemical structure 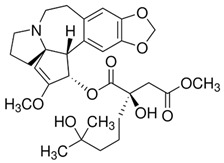			C_29_H_39_NO_9_ MW = 545.6 g/mol IUPAC name: 1-*O*-[(2*S*,3*S*,6*R*)-4-methoxy-16,18-dioxa-10-azapentacyclo[11.7.0.0^2,6^.0^6,10^.0^15,19^]icosa-1(20),4,13,15(19)-tetraen-3-yl] 4-*O*-methyl (2*R*)-2-hydroxy-2-(4-hydroxy-4-methylpentyl)butanedioate	

**Table 3:** Comparison of efficacy (cytotoxicity expressed by values of the IC_50_) and safety profile of alkaloids in various pharmacological models, highlighting the future perspectives for anti-glioblastoma therapies.

Alkaloids	Efficiency Expressed by Values of the IC_50_ (Cancer Cell Viability—Cytotoxicity)	Safety Profile	Perspectives for Anti-Glioblastoma Therapies
Berberin	IC_50_ = from 21.76 μmol/L to 42 μmol/L (U87), IC_50_ = from 9.79 µmol/L to 32 μmol/L (U251) [60], IC_50_ = 35.54 µmol/L (U118) [47]	- Favorable safety profile, observed only mild gastrointestinal adverse events in clinical trials [208] - Well-tolerated by the human body [209] - Low toxicity and no major adverse effects at standard doses [210]	- Clinical application can be severely limited by its unpleasant pharmacokinetic parameters, such as - Poor bioavailability - Limited absorption - Poor water solubility [211] - The novel pharmaceutic formulations using nanotechnology are needed
Boldine	IC_50_ = 68.6 μM (GBM59), IC_50_ = 213.8 μM (for U87-MG), IC_50_ = 141.7 μM (GBM96) [28]	- Good safety profile [212]	- Promising drug candidate to future clinical trials - Few results may have implications for future development of supportive treatment strategies for GM, including inhibition of proliferation of mutated neural stem cells or glioma stem cells that may remain in the brain after tumor resection [28] - Lacking the clinical trials
Brucine	IC_50_ = from 50 to 800 µmol/L (U251, U87, U118, A172)	- Toxic effects on nervous system (severe convulsions) immune system, urinary system, digestive system, cardiovascular system (increasing blood pressure), fatal poisoning [213]	- Serious toxicity may limit clinical applications [195] - The novel pharmaceutic formulations are needed to reduce side effects [213] - Lacking the clinical trials
Capsaicin	IC_50_ = from 265.7 μM to 325.7 μM (LN18) [69]	- Side effects: irritating the skin of humans, nausea, vomiting, abdominal pain, burning diarrhea [214] - Neurotoxic effects after systemic administration of high-dose [159]	- Nanoparticles with capsaicin may reduce adverse effects and can improve efficacy [215,216] - Capsaicin may be considered a potential therapeutic approach in anti-glioblastoma therapy - Lacking the clinical trials
Chelerythrine	IC_50_ = from 8 μM to 15 μM (U87, C6) [39]	- Significant weight loss of mice [120] - Decreasing the weight of spleens of the mice with obvious pathological lesions [120] - Any pathological changes in gastrointestinal system, kidney, liver, and heart observed in assessment of clinical toxicology in swine [217]	- Formulation of liposomes with chelerythrine modified with polyethylene glycol can reduce toxicity and may improve its anti-glioblastoma activity [120] - Lacking the clinical trials
Cocaine	IC_50_ = 6.76 mM (C6) [88]	- Neurotoxic potential in vitro and in vivo [218] - Cardiovascular toxicity: hypertension, tachycardia, cardiomyopathy, and acute myocardial infarction - Neurodegeneration, premature brain aging, depression, seizures, headaches, and hemorrhagic stroke (after chronic consumption) - Hepatotoxic effect - Acute kidney failure and rhabdomyolysis [201,218] - Downregulating brain microvascular GLUT1 (responsible for the reduced brain glucose uptake and cognitive impairment), leading to BBB dysfunction/leakage [219]	- Systematic preclinical and clinical studies on safety profile in patients with glioblastoma are needed - Lacking the clinical trials
Colchicine	IC_50_ = 10 nM (U87MG), IC_50_= 50 nM (U373MG) [84]	- Narrow therapeutic window and long half-life of elimination [139] - Gastrointestinal disorders, shock, progressive multiple organ failure, and myelosuppression [139]	- Colchicine was the most potent of microtubule-targeting agents in vitro in comparison with nocodazole, tivantinib, and CMPD1; however, colchicine incompletely kills glioblastoma stem cells and its anticancer activity is independent of tubulin isotypes and the post-translational modifications [77] - Lacking the clinical trials
Cyclopamine	IC_50_ = 11.4 μM (C6), IC_50_ = 7.7 μM (C6-GSCs) [82]	- Teratogenic potential in mice [220] - Teratogen caused the craniofacial birth defects (cyclops) in the offspring of sheep [221]	- More preclinical studies are needed - Lacking the clinical trials
Harmine	IC_50_ = 5 µM—50 µM (U251-MG and U373-MG) [40,41]	- Low neurotoxicity [222] - In healthy volunteers—doses > 2.7 mg/kg are associated with vomiting, drowsiness, and limited psychoactivity [223]	- Promising drug candidate to clinical trials [34] - Lacking the clinical trials
Kukoamine A	IC_50_ = 73.4 μg/mL (U251), IC_50_ = 22.1 μg/mL (WJ1), IC_50_ = 226.0 μg/mL (C6) [80]	- No adverse effects in mice [224],	- More preclinical studies are needed - Lacking the clinical trials
Lycorine	IC_50_ = 2.17 μM (in 229R), IC_50_ = 2.39 μM (in 251R) [72], IC_50_ = 2.85 μM (in C6), IC_50_ = 3.68 μM (U-87 MG) [14,74] IC_50_ = about 10 μM (U251) [73]	- Low toxicity and mild side effects in dogs and mice [188] - Nausea, emesis, diarrhea in dogs [188] - Maximum emetic dose of lycorine (ED100) was 2 mg/kg body weight in humans [188]	- Improving lipophilicity by formulation of lycorine–oleic acid nano-emulsion [192] - One hundred thirty-six common targets between lycorine and GBM [66] - Low toxicity positions them as promising candidates for anticancer drug development [162] - Lacking the long-term toxicity assessments of lycorine [162]
Nitidine	IC_50_ = 5.0 and 7.5 μM (U87) [38]	- Toxicity on liver, kidney, and heart with nitidine chloride, - Inhibit the proliferation of human embryonic kidney cell line - Cardiotoxic effect in zebrafish embryos [225]	- Poor solubility and low bioavailability [225] - New formulations as promising drug delivery systems with long-term and slow release: nitidine chloride–phospholipid complex and solid lipid nanoparticles with nitidine [225]
Noscapine	IC_50_ = from 85 to 131 μM [9]	- Exerting negligible toxicity, and non-addictive activity [9] - No toxic effects on peripheral axons and no induction of neuropathy [142]	- Analogs can be developed as new adjuvants to improve the efficacy of currently existing therapy - Noscapine deserves to be studied as a novel drug candidate against neurooncological diseases [83]
Piperine	IC_50_ = 120 µM (U87) [15], IC_50_ = 133 µM (T98G) [16]	- Administration by intravenous more toxic in comparison with intragastric, subcutaneously, and intramuscular routes [168] - Hemorrhagic ulceration of gastrointestinal tract [168] - Disturbance of spermatogenesis, maternal reproductive, embryotoxic effects in animal studies (after piperine bolus) [207], - Toxicity affects reproductive system [209] - Increasing in serum gonadotropins and decreasing in intratesticular testosterone (in rats) [226,227]	- Poor water solubility and low bioavailability [165] - Increasing effects of silybin [168,228] (flavonolignan with anti-glioblastoma activity by autophagy) [229] - Nanosuspension and emulosome as methods of bio-enhancing properties of piperine [168] - Limited number of clinical trials [168]
Tetrandrine	IC_50_ = 30.41 µM (U87), IC_50_ = 27.5 µM (U251 GSLCs) [45]	- Damage of liver and lung in animal models [230,231] - Several toxic side effects in humans [230,231]	- Side effects and toxicity limited clinical application as a drug [231] - Encapsulate tetrandrine can enhance its stability, and efficacy in cancer treatment [131] - Very limited studies on the safety, bioavailability, and pharmacokinetic parameters in clinical settings [131]

## Data Availability

The original contributions presented in the study are included in the article; further inquiries can be directed to the corresponding author.

## References

[1] Ostrom Q.T., Bauchet L., Davis F.G., Deltour I., Fisher J.L., Langer C.E., Pekmezci M., Schwartzbaum J.A., Turner M.C., Walsh K.M. (2014). The Epidemiology of Glioma in Adults: A “State of the Science” Review. Neuro Oncol..

[2] Patel A.P., Fisher J.L., Nichols E., Abd-Allah F., Abdela J., Abdelalim A., Abraha H.N., Agius D., Alahdab F., Alam T. (2019). Global, Regional, and National Burden of Brain and Other CNS Cancer, 1990–2016: A Systematic Analysis for the Global Burden of Disease Study 2016. Lancet Neurol..

[3] Grech N., Dalli T., Mizzi S., Meilak L., Calleja N., Zrinzo A. (2020). Rising Incidence of Glioblastoma Multiforme in a Well-Defined Population. Cureus.

[4] Kubelt C., Hattermann K., Sebens S., Mehdorn H.M., Held-Feindt J. (2015). Epithelial-to-Mesenchymal Transition in Paired Human Primary and Recurrent Glioblastomas. Int. J. Oncol..

[5] Rong L., Li N., Zhang Z. (2022). Emerging Therapies for Glioblastoma: Current State and Future Directions. J. Exp. Clin. Cancer Res..

[6] Kanderi T., Munakomi S., Gupta V. (2024). Glioblastoma Multiforme. StatPearls.

[7] Deo S.V.S., Sharma J., Kumar S. (2022). GLOBOCAN 2020 Report on Global Cancer Burden: Challenges and Opportunities for Surgical Oncologists. Ann. Surg. Oncol..

[8] Sung H., Ferlay J., Siegel R.L., Laversanne M., Soerjomataram I., Jemal A., Bray F. (2021). Global Cancer Statistics 2020: GLOBOCAN Estimates of Incidence and Mortality Worldwide for 36 Cancers in 185 Countries. CA A Cancer J. Clin..

[9] Lan Z., Li X., Zhang X. (2024). Glioblastoma: An Update in Pathology, Molecular Mechanisms and Biomarkers. Int. J. Mol. Sci..

[10] Shi X., Gekas C., Verduzco D., Petiwala S., Jeffries C., Lu C., Murphy E., Anton T., Vo A.H., Xiao Z. (2024). Building a Translational Cancer Dependency Map for The Cancer Genome Atlas. Nat. Cancer.

[11] General Principles of Cancer Chemotherapy. https://accesspharmacy.mhmedical.com/content.aspx?bookid=1810&sectionid=124496835.

[12] Bousbaa H. (2021). Novel Anticancer Strategies. Pharmaceutics.

[13] Matsuura H.N., Fett-Neto A.G., Gopalakrishnakone P., Carlini C.R., Ligabue-Braun R. (2015). Plant Alkaloids: Main Features, Toxicity, and Mechanisms of Action. Plant Toxins.

[14] Ali A.H., Abdelrahman M., El-Sayed M.A. (2019). Alkaloid Role in Plant Defense Response to Growth and Stress. Bioactive Molecules in Plant Defense.

[15] Olofinsan K., Abrahamse H., George B.P. (2023). Therapeutic Role of Alkaloids and Alkaloid Derivatives in Cancer Management. Molecules.

[16] Liu Q., Zhang C., Fang H., Yi L., Li M. (2023). Indispensable Biomolecules for Plant Defense against Pathogens: NBS-LRR and “Nitrogen Pool” Alkaloids. Plant Sci..

[17] Adamski Z., Blythe L.L., Milella L., Bufo S.A. (2020). Biological Activities of Alkaloids: From Toxicology to Pharmacology. Toxins.

[18] Karpiński T.M., Ożarowski M., Seremak-Mrozikiewicz A., Wolski H., Adamczak A. (2021). Plant Preparations and Compounds with Activities against Biofilms Formed by *Candida* Spp.. J. Fungi.

[19] Pereira A.G., Cassani L., Garcia-Oliveira P., Otero P., Mansoor S., Echave J., Xiao J., Simal-Gándara J., Prieto M.A., Carocho M., Heleno S.A., Barros L. (2023). Plant Alkaloids: Production, Extraction, and Potential Therapeutic Properties. Natural Secondary Metabolites: From Nature, Through Science, to Industry.

[20] Palma T.V., Lenz L.S., Bottari N.B., Pereira A., Schetinger M.R.C., Morsch V.M., Ulrich H., Pillat M.M., de Andrade C.M. (2020). Berberine Induces Apoptosis in Glioblastoma Multiforme U87MG Cells via Oxidative Stress and Independent of AMPK Activity. Mol. Biol. Rep..

[21] Rajput A., Sharma R., Bharti R. (2022). Pharmacological Activities and Toxicities of Alkaloids on Human Health. Mater. Today Proc..

[22] Aalinezhad S., Dabaghian F., Namdari A., Akaberi M., Emami S.A. (2024). Phytochemistry and Pharmacology of Alkaloids from Papaver Spp.: A Structure–Activity Based Study. Phytochem. Rev..

[23] Feng L.Y., Battulga A., Han E., Chung H., Li J.H. (2017). New psychoactive substances of natural origin: A brief review. J. Food Drug Anal..

[24] Benowitz N.L. (2009). Pharmacology of nicotine: Addiction, smoking-induced disease, and therapeutics. Annu. Rev. Pharmacol. Toxicol..

[25] Heinrich M., Mah J., Amirkia V. (2021). Alkaloids Used as Medicines: Structural Phytochemistry Meets Biodiversity-An Update and Forward Look. Molecules.

[26] Gonçalves J., Luís Â., Gallardo E., Duarte A.P. (2021). Psychoactive Substances of Natural Origin: Toxicological Aspects, Therapeutic Properties and Analysis in Biological Samples. Molecules.

[27] Devereaux A.L., Mercer S.L., Cunningham C.W. (2018). DARK Classics in Chemical Neuroscience: Morphine. ACS Chem. Neurosci..

[28] Jiménez-Madrona E., Morado-Díaz C.J., Talaverón R., Tabernero A., Pastor A.M., Sáez J.C., Matarredona E.R. (2023). Antiproliferative Effect of Boldine on Neural Progenitor Cells and on Glioblastoma Cells. Front. Neurosci..

[29] Zhu M., Niu J., Jiang J., Dong T., Chen Y., Yang X., Liu P. (2022). Chelerythrine Inhibits the Progression of Glioblastoma by Suppressing the TGFB1-ERK1/2/Smad2/3-Snail/ZEB1 Signaling Pathway. Life Sci..

[30] Silva T.C.C., de Faria Lopes G.P., Menezes-Filho N.d.J., de Oliveira D.M., Pereira E., Pitanga B.P.S., Dos Santos Costa R., da Silva Velozo E., Freire S.M., Clarêncio J. (2018). Specific Cytostatic and Cytotoxic Effect of Dihydrochelerythrine in Glioblastoma Cells: Role of NF-κB/β-Catenin and STAT3/IL-6 Pathways. Anticancer Agents Med. Chem..

[31] Jia M., Wang Y., Guo Y., Yu P., Sun Y., Song Y., Zhao L. (2021). Nitidine Chloride Suppresses Epithelial-Mesenchymal Transition and Stem Cell-like Properties in Glioblastoma by Regulating JAK2/STAT3 Signaling. Cancer Med..

[32] Wang P., Zheng S.-Y., Jiang R.-L., Wu H.-D., Li Y.-A., Lu J.-L., Xiong Y., Han B., Lin L. (2023). Necroptosis Signaling and Mitochondrial Dysfunction Cross-Talking Facilitate Cell Death Mediated by Chelerythrine in Glioma. Free Radic. Biol. Med..

[33] Liu S.-H., Wang Q.-Z., Liu T., Bai R., Ma M.-M., Liu Q.-L., Zhou H.-G., Liu J., Wang M. (2022). Enhanced Glioblastoma Selectivity of Harmine via the Albumin Carrier. J. Biomed. Nanotechnol..

[34] Zhu Y.-G., Lv Y.-X., Guo C.-Y., Xiao Z.-M., Jiang Q.-G., Kuang H., Zhang W.-H., Hu P. (2021). Harmine Inhibits the Proliferation and Migration of Glioblastoma Cells via the FAK/AKT Pathway. Life Sci..

[35] Vahedi M.M., Shahini A., Mottahedi M., Garousi S., Shariat Razavi S.A., Pouyamanesh G., Afshari A.R., Ferns G.A., Bahrami A. (2023). Harmaline Exerts Potentially Anti-Cancer Effects on U-87 Human Malignant Glioblastoma Cells in Vitro. Mol. Biol. Rep..

[36] Liao C.-L., Ma Y.-S., Hsia T.-C., Chou Y.-C., Lien J.-C., Peng S.-F., Kuo C.-L., Hsu F.-T. (2021). Tetrandrine Suppresses Human Brain Glioblastoma GBM 8401/Luc2 Cell-Xenografted Subcutaneous Tumors in Nude Mice In Vivo. Molecules.

[37] Jiang Y.-W., Cheng H.-Y., Kuo C.-L., Way T.-D., Lien J.-C., Chueh F.-S., Lin Y.-L., Chung J.-G. (2019). Tetrandrine Inhibits Human Brain Glioblastoma Multiforme GBM 8401 Cancer Cell Migration and Invasion in Vitro. Environ. Toxicol..

[38] Zhang Y., Wen Y.-L., Ma J.-W., Ye J.-C., Wang X., Huang J.-X., Meng C.-Y., Xu X.-Z., Wang S.-X., Zhong X.-Y. (2017). Tetrandrine Inhibits Glioma Stem-like Cells by Repressing β-Catenin Expression. Int. J. Oncol..

[39] Wang C., Liu X., Guo S. (2023). Network Pharmacology-Based Strategy to Investigate the Effect and Mechanism of α-Solanine against Glioma. BMC Complement. Med. Ther..

[40] Wang X., Zou S., Lan Y.-L., Xing J.-S., Lan X.-Q., Zhang B. (2017). Solasonine Inhibits Glioma Growth through Anti-Inflammatory Pathways. Am. J. Transl. Res..

[41] Liu Y., Chen Y., Zhu R., Xu L., Xie H.Q., Zhao B. (2021). Rutaecarpine Inhibits U87 Glioblastoma Cell Migration by Activating the Aryl Hydrocarbon Receptor Signaling Pathway. Front. Mol. Neurosci..

[42] Liu Q., Wang Q., Lv C., Liu Z., Gao H., Chen Y., Zhao G. (2021). Brucine Inhibits Proliferation of Glioblastoma Cells by Targeting the G-Quadruplexes in the c-Myb Promoter. J. Cancer.

[43] Jin Y., Zhang J., Pan Y., Shen W. (2022). Berberine Suppressed the Progression of Human Glioma Cells by Inhibiting the TGF-Β1/SMAD2/3 Signaling Pathway. Integr. Cancer Ther..

[44] Jin F., Xie T., Huang X., Zhao X. (2018). Berberine Inhibits Angiogenesis in Glioblastoma Xenografts by Targeting the VEGFR2/ERK Pathway. Pharm. Biol..

[45] Agnarelli A., Natali M., Garcia-Gil M., Pesi R., Tozzi M.G., Ippolito C., Bernardini N., Vignali R., Batistoni R., Bianucci A.M. (2018). Cell-Specific Pattern of Berberine Pleiotropic Effects on Different Human Cell Lines. Sci. Rep..

[46] Wang J., Qi Q., Feng Z., Zhang X., Huang B., Chen A., Prestegarden L., Li X., Wang J. (2016). Berberine Induces Autophagy in Glioblastoma by Targeting the AMPK/mTOR/ULK1-Pathway. Oncotarget.

[47] Liu Q., Xu X., Zhao M., Wei Z., Li X., Zhang X., Liu Z., Gong Y., Shao C. (2015). Berberine Induces Senescence of Human Glioblastoma Cells by Downregulating the EGFR-MEK-ERK Signaling Pathway. Mol. Cancer Ther..

[48] Bibak B., Shakeri F., Keshavarzi Z., Mollazadeh H., Javid H., Jalili-Nik M., Sathyapalan T., Afshari A.R., Sahebkar A. (2022). Anticancer Mechanisms of Berberine: A Good Choice for Glioblastoma Multiforme Therapy. Curr. Med. Chem..

[49] Och A., Podgórski R., Nowak R. (2020). Biological Activity of Berberine—A Summary Update. Toxins.

[50] Rauf A., Abu-Izneid T., Khalil A.A., Imran M., Shah Z.A., Emran T.B., Mitra S., Khan Z., Alhumaydhi F.A., Aljohani A.S.M. (2021). Berberine as a Potential Anticancer Agent: A Comprehensive Review. Molecules.

[51] Li N., Zhang P., Kiang K.M.Y., Cheng Y.S., Leung G.K.K. (2018). Caffeine Sensitizes U87-MG Human Glioblastoma Cells to Temozolomide through Mitotic Catastrophe by Impeding G2 Arrest. BioMed Res. Int..

[52] Cheng Y.-C., Ding Y.-M., Hueng D.-Y., Chen J.-Y., Chen Y. (2016). Caffeine Suppresses the Progression of Human Glioblastoma via Cathepsin B and MAPK Signaling Pathway. J. Nutr. Biochem..

[53] Liu J.-D., Song L.-J., Yan D.-J., Feng Y.-Y., Zang Y.-G., Yang Y. (2015). Caffeine Inhibits the Growth of Glioblastomas through Activating the Caspase-3 Signaling Pathway in Vitro. Eur. Rev. Med. Pharmacol. Sci..

[54] Jiang J., Lan Y.-Q., Zhang T., Yu M., Liu X.-Y., Li L.-H., Chen X.-P. (2015). The in Vitro Effects of Caffeine on Viability, Cycle Cycle Profiles, Proliferation, and Apoptosis of Glioblastomas. Eur. Rev. Med. Pharmacol. Sci..

[55] Bonafé G.A., Boschiero M.N., Sodré A.R., Ziegler J.V., Rocha T., Ortega M.M. (2021). Natural Plant Compounds: Does Caffeine, Dipotassium Glycyrrhizinate, Curcumin, and Euphol Play Roles as Antitumoral Compounds in Glioblastoma Cell Lines?. Front. Neurol..

[56] Pérez-Pérez D., Reyes-Vidal I., Chávez-Cortez E.G., Sotelo J., Magaña-Maldonado R. (2019). Methylxanthines: Potential Therapeutic Agents for Glioblastoma. Pharmaceuticals.

[57] Sun F., Han D.-F., Cao B.-Q., Wang B., Dong N., Jiang D.-H. (2016). Caffeine-Induced Nuclear Translocation of FoxO1 Triggers Bim-Mediated Apoptosis in Human Glioblastoma Cells. Tumour Biol..

[58] Inada M., Sato A., Shindo M., Yamamoto Y., Akasaki Y., Ichimura K., Tanuma S.-I. (2019). Anticancer Non-Narcotic Opium Alkaloid Papaverine Suppresses Human Glioblastoma Cell Growth. Anticancer Res..

[59] Inada M., Shindo M., Kobayashi K., Sato A., Yamamoto Y., Akasaki Y., Ichimura K., Tanuma S.-I. (2019). Anticancer Effects of a Non-Narcotic Opium Alkaloid Medicine, Papaverine, in Human Glioblastoma Cells. PLoS ONE.

[60] Kaiser S., Carvalho Â.R., Pittol V., Dietrich F., Manica F., Machado M.M., de Oliveira L.F.S., Oliveira Battastini A.M., Ortega G.G. (2016). Genotoxicity and Cytotoxicity of Oxindole Alkaloids from Uncaria Tomentosa (Cat’s Claw): Chemotype Relevance. J. Ethnopharmacol..

[61] Szoka L., Palka J. (2020). Capsaicin Up-Regulates pro-Apoptotic Activity of Thiazolidinediones in Glioblastoma Cell Line. Biomed. Pharmacother..

[62] Hacioglu C., Kar F. (2023). Capsaicin Induces Redox Imbalance and Ferroptosis through ACSL4/GPx4 Signaling Pathways in U87-MG and U251 Glioblastoma Cells. Metab. Brain Dis..

[63] Diaz-Vidal T., Armenta-Pérez V.P., Rosales-Rivera L.C., Basulto-Padilla G.C., Martínez-Pérez R.B., Mateos-Díaz J.C., Gutiérrez-Mercado Y.K., Canales-Aguirre A.A., Rodríguez J.A. (2024). Long Chain Capsaicin Analogues Synthetized by CALB-CLEAs Show Cytotoxicity on Glioblastoma Cell Lines. Appl. Microbiol. Biotechnol..

[64] Dong Q., Niu W., Mu M., Ye C., Wu P., Hu S., Niu C. (2024). Lycorine Hydrochloride Interferes with Energy Metabolism to Inhibit Chemoresistant Glioblastoma Multiforme Cell Growth through Suppressing PDK3. Mol. Cell. Biochem..

[65] Shen J., Zhang T., Cheng Z., Zhu N., Wang H., Lin L., Wang Z., Yi H., Hu M. (2018). Lycorine Inhibits Glioblastoma Multiforme Growth through EGFR Suppression. J. Exp. Clin. Cancer Res..

[66] Su J., Huo M., Xu F., Ding L. (2024). Molecular Mechanism of Lycorine in the Treatment of Glioblastoma Based on Network Pharmacology and Molecular Docking. Naunyn-Schmiedeberg’s Arch. Pharmacol..

[67] Wang R., Deng D., Shao N., Xu Y., Xue L., Peng Y., Liu Y., Zhi F. (2018). Evodiamine Activates Cellular Apoptosis through Suppressing PI3K/AKT and Activating MAPK in Glioma. Onco Targets Ther..

[68] Khan M., Bi Y., Qazi J.I., Fan L., Gao H. (2015). Evodiamine Sensitizes U87 Glioblastoma Cells to TRAIL via the Death Receptor Pathway. Mol. Med. Rep..

[69] Wu W.-S., Chien C.-C., Liu K.-H., Chen Y.-C., Chiu W.-T. (2017). Evodiamine Prevents Glioma Growth, Induces Glioblastoma Cell Apoptosis and Cell Cycle Arrest through JNK Activation. Am. J. Chin. Med..

[70] Dai Z., Wang L., Wang X., Zhao B., Zhao W., Bhardwaj S.S., Ye J., Yin Z., Zhang J., Zhao S. (2018). Oxymatrine Induces Cell Cycle Arrest and Apoptosis and Suppresses the Invasion of Human Glioblastoma Cells through the EGFR/PI3K/Akt/mTOR Signaling Pathway and STAT3. Oncol. Rep..

[71] Liu F., Wang B., Wang J., Ling X., Li Q., Meng W., Ma J. (2016). Oxymatrine Inhibits Proliferation and Migration While Inducing Apoptosis in Human Glioblastoma Cells. BioMed Res. Int..

[72] Wang Q., Li H., Sun Z., Dong L., Gao L., Liu C., Wang X. (2016). Kukoamine A Inhibits Human Glioblastoma Cell Growth and Migration through Apoptosis Induction and Epithelial-Mesenchymal Transition Attenuation. Sci. Rep..

[73] Bensalma S., Chadeneau C., Legigan T., Renoux B., Gaillard A., de Boisvilliers M., Pinet-Charvet C., Papot S., Muller J.M. (2015). Evaluation of Cytotoxic Properties of a Cyclopamine Glucuronide Prodrug in Rat Glioblastoma Cells and Tumors. J. Mol. Neurosci..

[74] Zhang L., Fu R., Duan D., Li Z., Li B., Ming Y., Li L., Ni R., Chen J. (2021). Cyclovirobuxine D Induces Apoptosis and Mitochondrial Damage in Glioblastoma Cells Through ROS-Mediated Mitochondrial Translocation of Cofilin. Front. Oncol..

[75] Fang K.-M., Liu J.-J., Li C.-C., Cheng C.-C., Hsieh Y.-T., Chai K.M., Lien Y.-A., Tzeng S.-F. (2015). Colchicine Derivative as a Potential Anti-Glioma Compound. J. Neuro-Oncol..

[76] Zottel A., Jovčevska I., Šamec N., Komel R. (2021). Cytoskeletal Proteins as Glioblastoma Biomarkers and Targets for Therapy: A Systematic Review. Crit. Rev. Oncol. Hematol..

[77] Abbassi R.H., Recasens A., Indurthi D.C., Johns T.G., Stringer B.W., Day B.W., Munoz L. (2019). Lower Tubulin Expression in Glioblastoma Stem Cells Attenuates Efficacy of Microtubule-Targeting Agents. ACS Pharmacol. Transl. Sci..

[78] Carone C., Genedani S., Leo G., Filaferro M., Fuxe K., Agnati L.F. (2015). In Vitro Effects of Cocaine on Tunneling Nanotube Formation and Extracellular Vesicle Release in Glioblastoma Cell Cultures. J. Mol. Neurosci..

[79] Steinmetz A., Steffens L., Morás A.M., Prezzi F., Braganhol E., Saffi J., Ortiz R.S., Barros H.M.T., Moura D.J. (2018). In Vitro Model to Study Cocaine and Its Contaminants. Chem. Biol. Interact..

[80] Park K.-J., Yu M.O., Park D.-H., Park J.-Y., Chung Y.-G., Kang S.-H. (2016). Role of Vincristine in the Inhibition of Angiogenesis in Glioblastoma. Neurol. Res..

[81] Wu M., Fan Y., Lv S., Xiao B., Ye M., Zhu X. (2016). Vincristine and Temozolomide Combined Chemotherapy for the Treatment of Glioma: A Comparison of Solid Lipid Nanoparticles and Nanostructured Lipid Carriers for Dual Drugs Delivery. Drug Deliv..

[82] Filippi-Chiela E.C., Vargas J.E., Bueno E., Silva M.M., Thomé M.P., Lenz G. (2022). Vincristine Promotes Differential Levels of Apoptosis, Mitotic Catastrophe, and Senescence Depending on the Genetic Background of Glioblastoma Cells. Toxicol. Vitr..

[83] Altinoz M.A., Topcu G., Hacimuftuoglu A., Ozpinar A., Ozpinar A., Hacker E., Elmaci İ. (2019). Noscapine, a Non-Addictive Opioid and Microtubule-Inhibitor in Potential Treatment of Glioblastoma. Neurochem. Res..

[84] Wang X., Zhuang Y., Wang Y., Jiang M., Yao L. (2023). The Recent Developments of Camptothecin and Its Derivatives as Potential Anti-Tumor Agents. Eur. J. Med. Chem..

[85] Mondal A., Gandhi A., Fimognari C., Atanasov A.G., Bishayee A. (2019). Alkaloids for Cancer Prevention and Therapy: Current Progress and Future Perspectives. Eur. J. Pharmacol..

[86] Turrini E., Sestili P., Fimognari C. (2020). Overview of the Anticancer Potential of the “King of Spices” Piper Nigrum and Its Main Constituent Piperine. Toxins.

[87] Jeong S., Jung S., Park G.-S., Shin J., Oh J.-W. (2020). Piperine Synergistically Enhances the Effect of Temozolomide against Temozolomide-Resistant Human Glioma Cell Lines. Bioengineered.

[88] Su J., Yin W., Huo M., Yao Q., Ding L. (2023). Induction of Apoptosis in Glioma Cells by Lycorine via Reactive Oxygen Species Generation and Regulation of NF-κB Pathways. Naunyn-Schmiedeberg’s Arch. Pharmacol..

[89] Warrier N.M., Krishnan R.K., Prabhu V., Hariharapura R.C., Agarwal P., Kumar P. (2022). Survivin Inhibition by Piperine Sensitizes Glioblastoma Cancer Stem Cells and Leads to Better Drug Response. Int. J. Mol. Sci..

[90] Diehl S., Hildebrandt G., Manda K. (2022). Pepper Alkaloid Piperine Increases Radiation Sensitivity of Cancer Cells from Glioblastoma and Hypopharynx In Vitro. Int. J. Mol. Sci..

[91] Nellan A., Wright E., Campbell K., Davies K.D., Donson A.M., Amani V., Judd A., Hemenway M.S., Raybin J., Foreman N.K. (2020). Retrospective Analysis of Combination Carboplatin and Vinblastine for Pediatric Low-Grade Glioma. J. Neuro-Oncol..

[92] Vairy S., Le Teuff G., Bautista F., De Carli E., Bertozzi A.-I., Pagnier A., Fouyssac F., Nysom K., Aerts I., Leblond P. (2020). Phase I Study of Vinblastine in Combination with Nilotinib in Children, Adolescents, and Young Adults with Refractory or Recurrent Low-Grade Glioma. Neuro-Oncol. Adv..

[93] Roux C., Revon-Rivière G., Gentet J.C., Verschuur A., Scavarda D., Saultier P., Appay R., Padovani L., André N. (2021). Metronomic Maintenance With Weekly Vinblastine After Induction With Bevacizumab-Irinotecan in Children With Low-Grade Glioma Prevents Early Relapse. J. Pediatr. Hematol. Oncol..

[94] Luo C., Ai J., Ren E., Li J., Feng C., Li X., Luo X. (2021). Research Progress on Evodiamine, a Bioactive Alkaloid of Evodiae Fructus: Focus on Its Anti-Cancer Activity and Bioavailability (Review). Exp. Ther. Med..

[95] Sarkaria J.N., Hu L.S., Parney I.F., Pafundi D.H., Brinkmann D.H., Laack N.N., Giannini C., Burns T.C., Kizilbash S.H., Laramy J.K. (2018). Is the Blood-Brain Barrier Really Disrupted in All Glioblastomas? A Critical Assessment of Existing Clinical Data. Neuro-Oncology.

[96] Carmustine Medac (Previously Carmustine Obvius)|European Medicines Agency (EMA). https://www.ema.europa.eu/en/medicines/human/EPAR/carmustine-medac.

[97] Temodal|European Medicines Agency (EMA). https://www.ema.europa.eu/en/medicines/human/EPAR/temodal.

[98] Zhou Y., Jia P., Fang Y., Zhu W., Gong Y., Fan T., Yin J. (2024). Comprehensive Understanding of the Adverse Effects Associated with Temozolomide: A Disproportionate Analysis Based on the FAERS Database. Front. Pharmacol..

[99] Wang L., Fei Y., Qu H., Zhang H., Wang Y., Wu Z., Fan G. (2024). Five Years of Safety Profile of Bevacizumab: An Analysis of Real-World Pharmacovigilance and Randomized Clinical Trials. J. Pharm. Health Care Sci..

[100] Majchrzak-Celińska A., Studzińska-Sroka E. (2024). New Avenues and Major Achievements in Phytocompounds Research for Glioblastoma Therapy. Molecules.

[101] Dey P., Kundu A., Chakraborty H.J., Kar B., Choi W.S., Lee B.M., Bhakta T., Atanasov A.G., Kim H.S. (2019). Therapeutic Value of Steroidal Alkaloids in Cancer: Current Trends and Future Perspectives. Int. J. Cancer.

[102] Tilaoui M., Ait Mouse H., Zyad A. (2021). Update and New Insights on Future Cancer Drug Candidates From Plant-Based Alkaloids. Front. Pharmacol..

[103] Cragg G.M., Newman D.J. (2000). Antineoplastic Agents from Natural Sources: Achievements and Future Directions. Expert Opin. Investig. Drugs.

[104] Varela C., Silva F., Costa G., Cabral C., Vitorino C., Balaña C., Cabral C. (2023). Chapter 1—Alkaloids: Their Relevance in Cancer Treatment. New Insights into Glioblastoma.

[105] Zishan M., Saidurrahman S., Anayatullah A., Azeemuddin A., Ahmad Z., Hussain M.W. (2017). Natural Products Used as Anti-Cancer Agents. J. Drug Deliv. Ther..

[106] Ferreira M.-J.U. (2022). Alkaloids in Future Drug Discovery. Molecules.

[107] Dhyani P., Quispe C., Sharma E., Bahukhandi A., Sati P., Attri D.C., Szopa A., Sharifi-Rad J., Docea A.O., Mardare I. (2022). Anticancer Potential of Alkaloids: A Key Emphasis to Colchicine, Vinblastine, Vincristine, Vindesine, Vinorelbine and Vincamine. Cancer Cell Int..

[108] Gach-Janczak K., Drogosz-Stachowicz J., Janecka A., Wtorek K., Mirowski M. (2024). Historical Perspective and Current Trends in Anticancer Drug Development. Cancers.

[109] Fuchs C., Mitchell E.P., Hoff P.M. (2006). Irinotecan in the Treatment of Colorectal Cancer. Cancer Treat. Rev..

[110] Lee D.H., Kim S.-W., Suh C., Lee J.-S., Lee J.H., Lee S.-J., Ryoo B.Y., Park K., Kim J.S., Heo D.S. (2008). Belotecan, New Camptothecin Analogue, Is Active in Patients with Small-Cell Lung Cancer: Results of a Multicenter Early Phase II Study. Ann. Oncol..

[111] McBain C., Lawrie T.A., Rogozińska E., Kernohan A., Robinson T., Jefferies S. (2021). Treatment Options for Progression or Recurrence of Glioblastoma: A Network Meta-Analysis. Cochrane Database Syst. Rev..

[112] Upadhyayula P.S., Spinazzi E.F., Argenziano M.G., Canoll P., Bruce J.N. (2020). Convection Enhanced Delivery of Topotecan for Gliomas: A Single-Center Experience. Pharmaceutics.

[113] Food and Drug Administration (2024). FDA Approves Irinotecan Liposome for First-Line Treatment of Metastatic Pancreatic Adenocarcinoma.

[114] O’Brien S., Kantarjian H., Keating M., Beran M., Koller C., Robertson L.E., Hester J., Rios M.B., Andreeff M., Talpaz M. (1995). Homoharringtonine Therapy Induces Responses in Patients with Chronic Myelogenous Leukemia in Late Chronic Phase. Blood.

[115] Leonard A., Wolff J.E. (2013). Etoposide Improves Survival in High-Grade Glioma: A Meta-Analysis. Anticancer Res..

[116] Mosca L., Ilari A., Fazi F., Assaraf Y.G., Colotti G. (2021). Taxanes in Cancer Treatment: Activity, Chemoresistance and Its Overcoming. Drug Resist. Updates.

[117] Rankovic Z. (2015). CNS Drug Design: Balancing Physicochemical Properties for Optimal Brain Exposure. J. Med. Chem..

[118] Schulz J.A., Hartz A.M.S., Bauer B. (2023). ABCB1 and ABCG2 Regulation at the Blood-Brain Barrier: Potential New Targets to Improve Brain Drug Delivery. Pharmacol. Rev..

[119] Abbott N.J., Patabendige A.A.K., Dolman D.E.M., Yusof S.R., Begley D.J. (2010). Structure and Function of the Blood-Brain Barrier. Neurobiol. Dis..

[120] Wang Y., Zhang F., Xiong N., Xu H., Chai S., Wang H., Wang J., Zhao H., Jiang X., Fu P. (2021). Remodelling and Treatment of the Blood-Brain Barrier in Glioma. Cancer Manag. Res..

[121] Noorani I., de la Rosa J. (2023). Breaking Barriers for Glioblastoma with a Path to Enhanced Drug Delivery. Nat. Commun..

[122] Patel M., Blaney S., Balis F.M., Grochow L.B., Ames M.M. (1998). Pharmacokinetics of drug delivery to the central nervous system. A Clinician’s Guide to Chemotherapy Pharmacokinetics and Pharmacodynamics.

[123] Wu D., Chen Q., Chen X., Han F., Chen Z., Wang Y. (2023). The blood–brain barrier: Structure, regulation, and drug delivery. Signal Transduct. Target. Ther..

[124] Pardridge W.M. (2005). The blood-brain barrier: Bottleneck in brain drug development. NeuroRx.

[125] Pardridge W.M. (2012). Drug transport across the blood-brain barrier. J. Cereb. Blood Flow. Metab..

[126] Mulvihill J.J., Cunnane E.M., Ross A.M., Duskey J.T., Tosi G., Grabrucker A.M. (2020). Drug delivery across the blood-brain barrier: Recent advances in the use of nanocarriers. Nanomedicine.

[127] Song X.-L., Liu S., Jiang Y., Gu L.-Y., Xiao Y., Wang X., Cheng L., Li X.-T. (2017). Targeting Vincristine plus Tetrandrine Liposomes Modified with DSPE-PEG2000-Transferrin in Treatment of Brain Glioma. Eur. J. Pharm. Sci..

[128] Lin J.-F., Liu Y.-S., Huang Y.-C., Chi C.-W., Tsai C.-C., Tsai T.-H., Chen Y.-J. (2022). Borneol and Tetrandrine Modulate the Blood-Brain Barrier and Blood-Tumor Barrier to Improve the Therapeutic Efficacy of 5-Fluorouracil in Brain Metastasis. Integr. Cancer Ther..

[129] Seelig A. (2020). P-Glycoprotein: One Mechanism, Many Tasks and the Consequences for Pharmacotherapy of Cancers. Front. Oncol..

[130] Zhang Y.-N., Yang Y.-F., Yang X.-W. (2018). Blood-Brain Barrier Permeability and Neuroprotective Effects of Three Main Alkaloids from the Fruits of Euodia Rutaecarpa with MDCK-pHaMDR Cell Monolayer and PC12 Cell Line. Biomed Pharmacother..

[131] Luan F., He X., Zeng N. (2020). Tetrandrine: A Review of Its Anticancer Potentials, Clinical Settings, Pharmacokinetics and Drug Delivery Systems. J. Pharm. Pharmacol..

[132] Iqbal S., Flux C., Briggs D.A., Deplazes E., Long J., Skrzypek R., Rothnie A., Kerr I.D., Callaghan R. (2022). Vinca Alkaloid Binding to P-Glycoprotein Occurs in a Processive Manner. Biochim. Biophys. Acta. Biomembr..

[133] Gai Y., Yang N., Chen J. (2020). Inhibitory Activity of 8 Alkaloids on P-Gp and Their Distribution in Chinese Uncaria Species. Nat. Prod. Commun..

[134] Joshi P., Vishwakarma R.A., Bharate S.B. (2017). Natural Alkaloids as P-Gp Inhibitors for Multidrug Resistance Reversal in Cancer. Eur. J. Med. Chem..

[135] Shah D., Ajazuddin, Bhattacharya S. (2023). Role of Natural P-Gp Inhibitor in the Effective Delivery for Chemotherapeutic Agents. J. Cancer Res. Clin. Oncol..

[136] El-Readi M.Z., Eid S., Abdelghany A.A., Al-Amoudi H.S., Efferth T., Wink M. (2019). Resveratrol Mediated Cancer Cell Apoptosis, and Modulation of Multidrug Resistance Proteins and Metabolic Enzymes. Phytomedicine.

[137] Dewanjee S., Dua T.K., Bhattacharjee N., Das A., Gangopadhyay M., Khanra R., Joardar S., Riaz M., Feo V.D., Zia-Ul-Haq M. (2017). Natural Products as Alternative Choices for P-Glycoprotein (P-Gp) Inhibition. Molecules.

[138] Ravikumar Reddy D., Khurana A., Bale S., Ravirala R., Samba Siva Reddy V., Mohankumar M., Godugu C. (2016). Natural Flavonoids Silymarin and Quercetin Improve the Brain Distribution of Co-Administered P-Gp Substrate Drugs. Springerplus.

[139] Wu J., Liu Z. (2022). Progress in the Management of Acute Colchicine Poisoning in Adults. Intern. Emerg. Med..

[140] Mehan S., Arora N., Bhalla S., Khan A., Rehman M.U., Alghamdi B.S., Zughaibi T.A., Ashraf G.M. (2022). Involvement of Phytochemical-Encapsulated Nanoparticles’ Interaction with Cellular Signalling in the Amelioration of Benign and Malignant Brain Tumours. Molecules.

[141] Alshammari M.K., Alghazwni M.K., Alharbi A.S., Alqurashi G.G., Kamal M., Alnufaie S.R., Alshammari S.S., Alshehri B.A., Tayeb R.H., Bougeis R.J.M. (2022). Nanoplatform for the Delivery of Topotecan in the Cancer Milieu: An Appraisal of Its Therapeutic Efficacy. Cancers.

[142] Hiser L., Herrington B., Lobert S. (2008). Effect of Noscapine and Vincristine Combination on Demyelination and Cell Proliferation in Vitro. Leuk. Lymphoma.

[143] Landen J.W., Hau V., Wang M., Davis T., Ciliax B., Wainer B.H., Van Meir E.G., Glass J.D., Joshi H.C., Archer D.R. (2004). Noscapine Crosses the Blood-Brain Barrier and Inhibits Glioblastoma Growth. Clin. Cancer Res..

[144] Li X.-T., Tang W., Xie H.-J., Liu S., Song X.-L., Xiao Y., Wang X., Cheng L., Chen G.-R. (2019). The Efficacy of RGD Modified Liposomes Loaded with Vinorelbine plus Tetrandrine in Treating Resistant Brain Glioma. J. Liposome Res..

[145] Ren T., Li M., Zheng H., Liu W., Zhang J. (2018). Microdialysis Combined with RRLC-MS/MS for the Pharmacokinetics of Two Major Alkaloids of Bi Qi Capsule and the Potential Roles of P-Gp and BCRP on Their Penetration. J. Chromatogr. B Analyt. Technol. Biomed. Life Sci..

[146] Zhao T., Zhang X., Zhao Y., Zhang L., Bai X., Zhang J., Zhao X., Chen L., Wang L., Cui L. (2014). Pretreatment by Evodiamine Is Neuroprotective in Cerebral Ischemia: Up-Regulated pAkt, pGSK3β, Down-Regulated NF-κB Expression, and Ameliorated BBB Permeability. Neurochem. Res..

[147] Hu Y., Yu X., Yang L., Xue G., Wei Q., Han Z., Chen H. (2024). Research Progress on the Antitumor Effects of Harmine. Front. Oncol..

[148] Miciaccia M., Rizzo F., Centonze A., Cavallaro G., Contino M., Armenise D., Baldelli O.M., Solidoro R., Ferorelli S., Scarcia P. (2024). Harmaline to Human Mitochondrial Caseinolytic Serine Protease Activation for Pediatric Diffuse Intrinsic Pontine Glioma Treatment. Pharmaceuticals.

[149] Zetler G., Back G., Iven H. (1974). Pharmacokinetics in the Rat of the Hallucinogenic Alkaloids Harmine and Harmaline. Naunyn-Schmiedeberg’s Arch. Pharmacol..

[150] Cisternino S., Rousselle C., Debray M., Scherrmann J.-M. (2003). In Vivo Saturation of the Transport of Vinblastine and Colchicine by P-Glycoprotein at the Rat Blood-Brain Barrier. Pharm. Res..

[151] Drion N., Lemaire M., Lefauconnier J.M., Scherrmann J.M. (1996). Role of P-Glycoprotein in the Blood-Brain Transport of Colchicine and Vinblastine. J. Neurochem..

[152] Pranata R., Feraldho A., Lim M.A., Henrina J., Vania R., Golden N., July J. (2022). Coffee and Tea Consumption and the Risk of Glioma: A Systematic Review and Dose-Response Meta-Analysis. Br. J. Nutr..

[153] Eigenmann D.E., Dürig C., Jähne E.A., Smieško M., Culot M., Gosselet F., Cecchelli R., Helms H.C.C., Brodin B., Wimmer L. (2016). In Vitro Blood-Brain Barrier Permeability Predictions for GABAA Receptor Modulating Piperine Analogs. Eur. J. Pharm. Biopharm..

[154] Han Y., Chin Tan T.M., Lim L.-Y. (2008). In Vitro and in Vivo Evaluation of the Effects of Piperine on P-Gp Function and Expression. Toxicol. Appl. Pharmacol..

[155] Jiang Z., Wang X., Zhang Y., Zhao P., Luo Z., Li J. (2015). Effect of Capsaicin-Loading Nanoparticles on Gliomas. J. Nanosci. Nanotechnol..

[156] Weaver T., Nogrady D.F. (2005). Medicinal chemistry. A Molecular and Biochemical Approach.

[157] Talevi A. (2021). Central Nervous System Multiparameter Optimization Desirability. The ADME Encyclopedia.

[158] Stéen E.J.L., Vugts D.J., Windhorst A.D. (2022). The Application of in silico Methods for Prediction of Blood-Brain Barrier Permeability of Small Molecule PET Tracers. Front. Nucl. Med..

[159] Isabel U.-V., de la Belén A., Riera M., Serrano Dolores R., Elena G.-B. (2024). A new frontier in neuropharmacology: Recent progress in natural products research for blood–brain barrier crossing. Curr. Res. Biotechnol..

[160] Alsabri Sami G., Mari Walid O., Sara Y., Alsadawi Murad A., Oroszi Terry L. (2018). Kinetic and Dynamic Description of Caffeine. J. Caffeine Adenosine Res..

[161] Ikeda-Murakami K., Tani N., Ikeda T., Aoki Y., Ishikawa T. (2022). Central Nervous System Stimulants Limit Caffeine Transport at the Blood-Cerebrospinal Fluid Barrier. Int. J. Mol. Sci..

[162] Zhang M., Zhang H., Jia L., Zhang Y., Qin R., Xu S., Mei Y. (2024). Health Benefits and Mechanisms of Theobromine. J. Funct. Foods.

[163] Sugimoto N., Katakura M., Matsuzaki K., Ohno-Shosaku T., Yachie A., Shido O. (2016). Theobromine, the Primary Methylxanthine Found in Theobroma Cacao, Can Pass through the Blood-Brain Barrier in Mice. FASEB J..

[164] Sugimoto N., Miwa S., Hitomi Y., Nakamura H., Tsuchiya H., Yachie A. (2014). Theobromine, the Primary Methylxanthine Found in Theobroma Cacao, Prevents Malignant Glioblastoma Proliferation by Negatively Regulating Phosphodiesterase-4, Extracellular Signal-Regulated Kinase, Akt/Mammalian Target of Rapamycin Kinase, and Nuclear Factor-Kappa, B. Nutr. Cancer.

[165] Judelson D.A., Preston A.G., Miller D.L., Muñoz C.X., Kellogg M.D., Lieberman H.R. (2013). Effects of Theobromine and Caffeine on Mood and Vigilance. J. Clin. Psychopharmacol..

[166] He D., Wu H., Wei Y., Liu W., Huang F., Shi H., Zhang B., Wu X., Wang C. (2015). Effects of Harmine, an Acetylcholinesterase Inhibitor, on Spatial Learning and Memory of APP/PS1 Transgenic Mice and Scopolamine-Induced Memory Impairment Mice. Eur. J. Pharmacol..

[167] Srinivasan K. (2007). Black Pepper and Its Pungent Principle-Piperine: A Review of Diverse Physiological Effects. Crit. Rev. Food Sci. Nutr..

[168] Tripathi A.K., Ray A.K., Mishra S.K. (2022). Molecular and Pharmacological Aspects of Piperine as a Potential Molecule for Disease Prevention and Management: Evidence from Clinical Trials. Beni. Suef. Univ. J. Basic Appl. Sci..

[169] Dubey R.K., Leeners B., Imthurn B., Merki-Feld G.S., Rosselli M. (2017). Piperine Decreases Binding of Drugs to Human Plasma and Increases Uptake by Brain Microvascular Endothelial Cells. Phytother. Res..

[170] Thornton T., Mills D., Bliss E. (2023). Capsaicin: A Potential Treatment to Improve Cerebrovascular Function and Cognition in Obesity and Ageing. Nutrients.

[171] Inyang D., Saumtally T., Nnadi C.N., Devi S., So P.-W. (2023). A Systematic Review of the Effects of Capsaicin on Alzheimer’s Disease. Int. J. Mol. Sci..

[172] Saria A., Skofitsch G., Lembeck F. (1982). Distribution of Capsaicin in Rat Tissues after Systemic Administration. J. Pharm. Pharmacol..

[173] 173 O’Neill J., Brock C., Olesen A.E., Andresen T., Nilsson M., Dickenson A.H. (2012). Unravelling the Mystery of Capsaicin: A Tool to Understand and Treat Pain. Pharmacol. Rev..

[174] Pasierski M., Szulczyk B. (2022). Beneficial Effects of Capsaicin in Disorders of the Central Nervous System. Molecules.

[175] Donnerer J., Amann R., Schuligoi R., Lembeck F. (1990). Absorption and Metabolism of Capsaicinoids Following Intragastric Administration in Rats. Naunyn-Schmiedeberg’s Arch. Pharmacol..

[176] Abdel-Salam O.M.E., Mózsik G. (2023). Capsaicin, The Vanilloid Receptor TRPV1 Agonist in Neuroprotection: Mechanisms Involved and Significance. Neurochem. Res..

[177] Wang H., Tao Z., Feng M., Li X., Deng Z., Zhao G., Yin H., Pan T., Chen G., Feng Z. (2020). Dual PLK1 and STAT3 Inhibition Promotes Glioblastoma Cells Apoptosis through MYC. Biochem. Biophys. Res. Commun..

[178] Bhattacharjee A.K., Kondoh T., Nagashima T., Ikeda M., Ehara K., Tamaki N. (2001). Quantitative Analysis of Papaverine-Mediated Blood-Brain Barrier Disruption in Rats. Biochem. Biophys. Res. Commun..

[179] Xu D.-H., Yan M., Li H.-D., Fang P.-F., Liu Y.-W. (2012). Influence of P-Glycoprotein on Brucine Transport at the in Vitro Blood-Brain Barrier. Eur. J. Pharmacol..

[180] Torres-Vega J., Gómez-Alonso S., Pérez-Navarro J., Pastene-Navarrete E. (2020). Green Extraction of Alkaloids and Polyphenols from Peumus Boldus Leaves with Natural Deep Eutectic Solvents and Profiling by HPLC-PDA-IT-MS/MS and HPLC-QTOF-MS/MS. Plants.

[181] Kazemi Noureini S., Tanavar F. (2015). Boldine, a Natural Aporphine Alkaloid, Inhibits Telomerase at Non-Toxic Concentrations. Chem. Biol. Interact..

[182] Pennisi G., Bruzzaniti P., Burattini B., Piaser Guerrato G., Della Pepa G.M., Sturiale C.L., Lapolla P., Familiari P., La Pira B., D’Andrea G. (2024). Advancements in Telomerase-Targeted Therapies for Glioblastoma: A Systematic Review. Int. J. Mol. Sci..

[183] Sun Y., Huang H., Zhan Z., Gao H., Zhang C., Lai J., Cao J., Li C., Chen Y., Liu Z. (2022). Berberine Inhibits Glioma Cell Migration and Invasion by Suppressing TGF-Β1/COL11A1 Pathway. Biochem. Biophys. Res. Commun..

[184] Liu Z., Chen Y., Gao H., Xu W., Zhang C., Lai J., Liu X., Sun Y., Huang H. (2020). Berberine Inhibits Cell Proliferation by Interfering with Wild-Type and Mutant P53 in Human Glioma Cells. Onco Targets Ther..

[185] Chen N., Qi Y., Ma X., Xiao X., Liu Q., Xia T., Xiang J., Zeng J., Tang J. (2022). Rediscovery of Traditional Plant Medicine: An Underestimated Anticancer Drug of Chelerythrine. Front. Pharmacol..

[186] Golán-Cancela I., Caja L. (2024). The TGF-β Family in Glioblastoma. Int. J. Mol. Sci..

[187] Fu W., Hou X., Dong L., Hou W. (2023). Roles of STAT3 in the Pathogenesis and Treatment of Glioblastoma. Front. Cell Dev. Biol..

[188] Luwor R.B., Stylli S.S., Kaye A.H. (2013). The Role of Stat3 in Glioblastoma Multiforme. J. Clin. Neurosci..

[189] Cao Z., Yang P., Zhou Q. (2013). Multiple Biological Functions and Pharmacological Effects of Lycorine. Sci. China Chem..

[190] Kuo Y.-H., Chan T.-C., Lai H.-Y., Chen T.-J., Wu L.-C., Hsing C.-H., Li C.-F. (2021). Overexpression of Pyruvate Dehydrogenase Kinase-3 Predicts Poor Prognosis in Urothelial Carcinoma. Front. Oncol..

[191] Liu Z., Li L., Liu L., Zhu Z., Yu Y., Zhan S., Luo Z., Li Y., Long H., Cai J. (2024). PDK3 Drives Colorectal Carcinogenesis and Immune Evasion and Is a Therapeutic Target for Boosting Immunotherapy. Am. J. Cancer Res..

[192] Xie Z., Li X., Chen H., Zeng A., Shi Y., Tang Y. (2019). The lncRNA-DLEU2/miR-186-5p/PDK3 Axis Promotes the Progress of Glioma Cells. Am. J. Transl. Res..

[193] Roy M., Liang L., Xiao X., Feng P., Ye M., Liu J. (2018). Lycorine: A Prospective Natural Lead for Anticancer Drug Discovery. Biomed. Pharmacother..

[194] Soleimani A., Rahmani F., Ferns G.A., Ryzhikov M., Avan A., Hassanian S.M. (2020). Role of the NF-κB Signaling Pathway in the Pathogenesis of Colorectal Cancer. Gene.

[195] Sahukari R., Punabaka J., Bhasha S., Ganjikunta V.S., Ramudu S.K., Kesireddy S.R. (2020). Plant Compounds for the Treatment of Diabetes, a Metabolic Disorder: NF-κB as a Therapeutic Target. Curr. Pharm. Des..

[196] Khabibov M., Garifullin A., Boumber Y., Khaddour K., Fernandez M., Khamitov F., Khalikova L., Kuznetsova N., Kit O., Kharin L. (2022). Signaling Pathways and Therapeutic Approaches in Glioblastoma Multiforme (Review). Int. J. Oncol..

[197] Ji J., Ding K., Luo T., Zhang X., Chen A., Zhang D., Li G., Thorsen F., Huang B., Li X. (2021). TRIM22 Activates NF-κB Signaling in Glioblastoma by Accelerating the Degradation of IκBα. Cell Death Differ..

[198] Parasramka S., Talari G., Rosenfeld M., Guo J., Villano J.L. (2017). Procarbazine, Lomustine and Vincristine for Recurrent High-Grade Glioma. Cochrane Database Syst. Rev..

[199] Chen Y., Chen J.-C., Tseng S.-H. (2009). Tetrandrine Suppresses Tumor Growth and Angiogenesis of Gliomas in Rats. Int. J. Cancer.

[200] Manoranjan B., Provias J.P. (2022). β-Catenin Marks Proliferating Endothelial Cells in Glioblastoma. J. Clin. Neurosci..

[201] Guo R., Wang T., Zhou G., Xu M., Yu X., Zhang X., Sui F., Li C., Tang L., Wang Z. (2018). Botany, Phytochemistry, Pharmacology and Toxicity of *Strychnos Nux-Vomica* L.: A Review. Am. J. Chin. Med..

[202] Senrung A., Tripathi T., Yadav J., Janjua D., Chaudhary A., Chhokar A., Aggarwal N., Joshi U., Goswami N., Bharti A.C. (2023). In Vivo Antiangiogenic Effect of Nimbolide, Trans-Chalcone and Piperine for Use against Glioblastoma. BMC Cancer.

[203] Javed B., Zhao X., Cui D., Curtin J., Tian F. (2021). Enhanced Anticancer Response of Curcumin- and Piperine-Loaded Lignin-g-p (NIPAM-Co-DMAEMA) Gold Nanogels against U-251 MG Glioblastoma Multiforme. Biomedicines.

[204] Xia L.-Y., Zhang Y.-L., Yang R., Wang Z.-C., Lu Y.-D., Wang B.-Z., Zhu H.-L. (2020). Tubulin Inhibitors Binding to Colchicine-Site: A Review from 2015 to 2019. Curr. Med. Chem..

[205] Nandre R.M., Terse P.S. (2025). An overview of immunotoxicity in drug discovery and development. Toxicol. Lett..

[206] Singh N., Vayer P., Tanwar S., Poyet J.-L., Tsaioun K., Villoutreix B.O. (2023). Drug discovery and development: Introduction to the general public and patient groups. Front. Drug. Discov..

[207] Paul D., Sanap G., Shenoy S., Kalyane D., Kalia K., Tekade R.K. (2021). Artificial intelligence in drug discovery and development. Drug Discov. Today.

[208] Ye Y., Liu X., Wu N., Han Y., Wang J., Yu Y., Chen Q. (2021). Efficacy and Safety of Berberine Alone for Several Metabolic Disorders: A Systematic Review and Meta-Analysis of Randomized Clinical Trials. Front. Pharmacol..

[209] Nie Q., Li M., Huang C., Yuan Y., Liang Q., Ma X., Qiu T., Li J. (2024). The Clinical Efficacy and Safety of Berberine in the Treatment of Non-Alcoholic Fatty Liver Disease: A Meta-Analysis and Systematic Review. J. Transl. Med..

[210] Lau Y.S., Ling W.C., Murugan D., Mustafa M.R. (2015). Boldine Ameliorates Vascular Oxidative Stress and Endothelial Dysfunction: Therapeutic Implication for Hypertension and Diabetes. J. Cardiovasc. Pharmacol..

[211] Behl T., Singh S., Sharma N., Zahoor I., Albarrati A., Albratty M., Meraya A.M., Najmi A., Bungau S. (2022). Expatiating the Pharmacological and Nanotechnological Aspects of the Alkaloidal Drug Berberine: Current and Future Trends. Molecules.

[212] Ruijun W., Wenbin M., Yumin W., Ruijian Z., Puweizhong H., Yulin L. (2014). Inhibition of Glioblastoma Cell Growth In Vitro and In Vivo by Brucine, a Component of Chinese Medicine. Oncol. Res..

[213] Lu L., Huang R., Wu Y., Jin J.-M., Chen H.-Z., Zhang L.-J., Luan X. (2020). Brucine: A Review of Phytochemistry, Pharmacology, and Toxicology. Front. Pharmacol..

[214] Huang Z., Sharma M., Dave A., Yang Y., Chen Z.-S., Radhakrishnan R. (2022). The Antifibrotic and the Anticarcinogenic Activity of Capsaicin in Hot Chili Pepper in Relation to Oral Submucous Fibrosis. Front. Pharmacol..

[215] Zhang W., Zhang Y., Fan J., Feng Z., Song X. (2024). Pharmacological Activity of Capsaicin: Mechanisms and Controversies (Review). Mol. Med. Rep..

[216] Yue W.W.S., Yuan L., Braz J.M., Basbaum A.I., Julius D. (2022). TRPV1 Drugs Alter Core Body Temperature via Central Projections of Primary Afferent Sensory Neurons. Elife.

[217] Kosina P., Walterová D., Ulrichová J., Lichnovský V., Stiborová M., Rýdlová H., Vicar J., Krecman V., Brabec M.J., Simánek V. (2004). Sanguinarine and Chelerythrine: Assessment of Safety on Pigs in Ninety Days Feeding Experiment. Food Chem. Toxicol..

[218] Roque Bravo R., Faria A.C., Brito-da-Costa A.M., Carmo H., Mladěnka P., Dias da Silva D., Remião F., On Behalf of the Oemonom Researchers (2022). Cocaine: An Updated Overview on Chemistry, Detection, Biokinetics, and Pharmacotoxicological Aspects Including Abuse Pattern. Toxins.

[219] Wei J., Liu H., Li Y., Zhao D., Wang B., Wang H., Wang L., Wang K., Yue J., Zhang H. (2024). Melatonin Protects Against Cocaine-Induced Blood−Brain Barrier Dysfunction and Cognitive Impairment by Regulating miR-320a-Dependent GLUT1 Expression. J. Pineal Res..

[220] Lipinski R.J., Hutson P.R., Hannam P.W., Nydza R.J., Washington I.M., Moore R.W., Girdaukas G.G., Peterson R.E., Bushman W. (2008). Dose- and Route-Dependent Teratogenicity, Toxicity, and Pharmacokinetic Profiles of the Hedgehog Signaling Antagonist Cyclopamine in the Mouse. Toxicol. Sci..

[221] Lee S.T., Welch K.D., Panter K.E., Gardner D.R., Garrossian M., Chang C.-W.T. (2014). Cyclopamine: From Cyclops Lambs to Cancer Treatment. J. Agric. Food Chem..

[222] Zhang L., Li D., Yu S. (2020). Pharmacological Effects of Harmine and Its Derivatives: A Review. Arch. Pharm. Res..

[223] Ables J.L., Israel L., Wood O., Govindarajulu U., Fremont R.T., Banerjee R., Liu H., Cohen J., Wang P., Kumar K. (2024). A Phase 1 Single Ascending Dose Study of Pure Oral Harmine in Healthy Volunteers. J. Psychopharmacol..

[224] Butts C.A., Hedderley D.I., Martell S., Dinnan H., Middlemiss-Kraak S., Bunn B.J., McGhie T.K., Lill R.E. (2022). Influence of Oral Administration of Kukoamine A on Blood Pressure in a Rat Hypertension Model. PLoS ONE.

[225] Lu Q., Luo S., Shi Z., Yu M., Guo W., Li C. (2022). Nitidine Chloride, a Benzophenanthridine Alkaloid from Zanthoxylum Nitidum (Roxb.) DC., Exerts Multiple Beneficial Properties, Especially in Tumors and Inflammation-Related Diseases. Front. Pharmacol..

[226] Daware M.B., Mujumdar A.M., Ghaskadbi S. (2000). Reproductive Toxicity of Piperine in Swiss Albino Mice. Planta Med..

[227] Malini T., Manimaran R.R., Arunakaran J., Aruldhas M.M., Govindarajulu P. (1999). Effects of Piperine on Testis of Albino Rats. J. Ethnopharmacol..

[228] Bi X., Yuan Z., Qu B., Zhou H., Liu Z., Xie Y. (2019). Piperine Enhances the Bioavailability of Silybin via Inhibition of Efflux Transporters BCRP and MRP2. Phytomedicine.

[229] Bai Z.-L., Tay V., Guo S.-Z., Ren J., Shu M.-G. (2018). Silibinin Induced Human Glioblastoma Cell Apoptosis Concomitant with Autophagy through Simultaneous Inhibition of mTOR and YAP. BioMed Res. Int..

[230] Liu T., Liu X., Li W. (2016). Tetrandrine, a Chinese Plant-Derived Alkaloid, Is a Potential Candidate for Cancer Chemotherapy. Oncotarget.

[231] Schütz R., Müller M., Geisslinger F., Vollmar A., Bartel K., Bracher F. (2020). Synthesis, Biological Evaluation and Toxicity of Novel Tetrandrine Analogues. Eur. J. Med. Chem..

[232] Committee for Medicinal Products for Human Use (CHMP) (2023). Guideline on the Clinical Evaluation of Anticancer Medicinal Products.

